# Satellite cell attrition and skeletal muscle aging

**DOI:** 10.1038/s41413-026-00596-4

**Published:** 2026-11-13

**Authors:** Yujia Teng, Ying Yao, Zhihang Zuo, Yuwei Li, Binxu Yin, Qian Wang, Kun Zhang

**Affiliations:** 1 Department of Rehabilitation Medicine, Sichuan Academy of Medical Sciences, Sichuan Provincial People’s Hospital, School of Medicine, University of Electronic Science and Technology of China, Chengdu, China; 2 Department of Pharmacy and Ultrasound Medicine and Computational Cardiology Key Laboratory of Sichuan Province, Sichuan Provincial People’s Hospital, Chengdu, China; 3 Shanghai Operating Room, Tongji Hospital, School of Medicine, Tongji University, Shanghai, China

**Keywords:** Metabolic syndrome, Homeostasis

## Abstract

Skeletal muscle aging involves the progressive decline in mass and function of muscle tissue, significantly impacting mobility and quality of life in older adults. Central to this process is the functional deterioration of SCs, the primary muscle stem cells responsible for regeneration. Aging impairs SC self-renewal, activation, proliferation, and differentiation through mechanisms including telomere attrition, mitochondrial dysfunction, epigenetic dysregulation, and aberrant signaling pathways such as Notch/Wnt and TGF-β. Additionally, the aged microenvironment—marked by chronic inflammation, oxidative stress, fibro-adipogenic infiltration, immune dysregulation, and vascular decline—further suppresses SC activity. Recent advances in multidisciplinary research, encompassing single-cell genomics, epigenetics, and bioengineering, have deepened our understanding of these mechanisms. Emerging interventions, such as nutritional strategies, exercise, pharmacological agents, cell therapy, and gene editing, offer promising avenues for restoring SC function and rejuvenating muscle regeneration. Future efforts should focus on integrated, personalized approaches to combat sarcopenia and improve healthspan.

## Introduction

Skeletal muscle is striated muscle tissue attached to bones via tendons and innervated by somatic motor nerves. It plays a vital role in maintaining posture and motor function, and also acts as an active endocrine organ that regulates systemic metabolic homeostasis by secreting multiple myokines. However, skeletal muscle undergoes progressive decline in both mass and function with advancing age—a pathological state defined as sarcopenia.^1^ Sarcopenia is characterized by reduced muscle strength, diminished mobility, impaired balance, and an increased risk of falls, severely impacting the quality of life and overall health of older adults. The World Health Organization (WHO) has classified it as an independent disease entity.

The core mechanism of skeletal muscle aging involves the functional decline and reduced number of satellite cells (SCs). These myogenic stem cells are located between the basement membrane and sarcolemma of muscle fibers and play a pivotal role in muscle regeneration. During aging, their self-renewal capacity significantly declines due to increased expression of cell cycle inhibitors, such as p21Cip1 (Cdkn1a) and p57Kip2 (Cdkn1c), leading to proliferation arrest.^2^ Concurrently, key regulators of myogenic differentiation (e.g., MyoD and Myf5) decrease, while inhibitory factors (e.g., Id3 and Runx1) increase, thereby impeding differentiation into mature muscle fibers.^3^ Single-cell transcriptomics reveals that aged SCs exhibit a “differentiation arrest” state and highly express fibrosis-associated genes (e.g., Col1a1).^4^ Mitochondrial dysfunction and dysregulated autophagy further exacerbate functional decline, whereas autophagy induction (e.g., via Tat-Beclin1) partially restores regenerative capacity.^5^ Additionally, senescent SCs secrete the S100B protein, which promotes muscle atrophy by activating the RAGE signaling pathway;^6^ the angiotensin-like protein ANGPTL2 inhibits self-renewal, creating a vicious cycle.^7^ Recent studies suggest that ferroptosis may accelerate SC senescence through iron ion accumulation mediated by the transferrin receptor (Tfr1).^8^

The deterioration of the aging microenvironment exacerbates satellite cell attrition and skeletal muscle functional decline. The paracrine function of fibroadipogenic progenitor cells (FAPs) diminishes with reduced secretion of growth differentiation factor 10 (GDF10) and insulin-like growth factor 1 (IGF-1), weakening myogenic signaling.^5^ The senescence-associated secretory phenotype (SASP) and chronic low-grade inflammation suppress SC activity through proinflammatory factors such as TNF-α and IFN-γ, with M1 macrophages and senescent T cells playing pivotal roles in this process.^9,10^ Alterations in the extracellular matrix (ECM) are also significant. FAPs abnormally differentiate into fibroblasts or adipocytes, leading to collagen deposition and ECM stiffening, which inhibits satellite cell activation,^11^ and disrupts myogenesis via the YAP/TAZ signaling pathway.^12^ Furthermore, neuromuscular junction (NMJ) degeneration and diminished motor neuron innervation promote the conversion of fast-twitch to slow-twitch fibers, impairing contractile function.^13,14^ Schwann cell dysfunction and reduced neurotrophic factor secretion exacerbate this process.^15^ Vascular sparseness and endothelial dysfunction induce local ischemia and hypoxia, limiting nutrient and oxygen supply.^16,17^ Microenvironmental factors, such as mechanical stimulation, hypoxia, and inflammation, also regulate myocyte metabolism, repair, and regeneration through molecular mechanisms involving Piezo1, HIF-1α, AMPK/mTOR, and immune cell polarization.^11,18–20^

In recent years, research on skeletal muscle aging and regeneration has exhibited increasing multidisciplinary convergence. Collaborative innovation across fields such as cell biology, molecular genetics, bioengineering, and computational biology has significantly advanced our understanding of the mechanisms underlying satellite cell homeostasis and aging. The integrated application of cutting-edge technologies, including single-cell sequencing, gene editing, stem cell-induced differentiation, and biomaterial engineering, has overcome scientific bottlenecks previously inaccessible to conventional methods. These approaches enable investigation across scales—from microscopic epigenetic regulation and signaling pathway networks to macroscopic muscle tissue structure and functional decline—comprehensively enhancing our understanding of satellite cell behavior and microenvironmental evolution during skeletal muscle aging. They also lay a solid foundation for developing targeted, personalized intervention strategies. This review outlines key mechanisms in satellite cell aging and recent advances in intervention strategies, focusing on the innovative potential of interdisciplinary approaches in advancing muscle regeneration research.

## Biological characteristics of satellite cells

### Identification and molecular markers of satellite cells

Satellite cells (SCs), also known as muscle stem cells (MuSCs), are a critical type of adult stem cell in skeletal muscle. Located between the basement membrane and the sarcolemma of muscle fibers, SCs play a central role in maintaining muscle homeostasis, repairing damage, and facilitating regeneration. Under normal conditions, SCs remain quiescent, but upon stimulation by injury, exercise, or disease, they activate and enter a proliferative phase, differentiating into myoblasts that ultimately fuse to form new muscle fibers or replenish the stem cell pool.

**Fig. 1:**
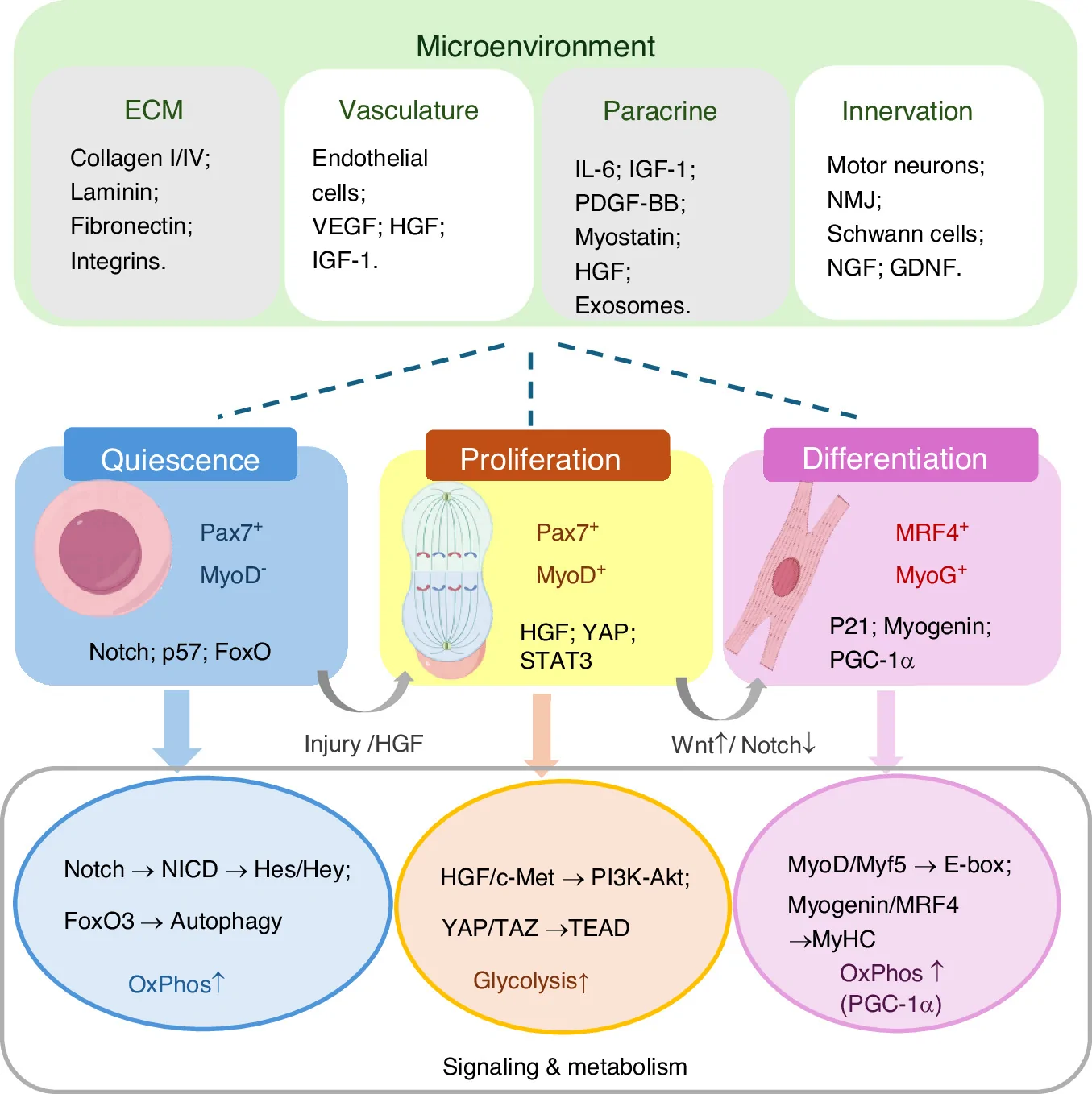
Biological characteristics of satellite cells. Schematic of skeletal muscle satellite cells and their microenvironment, illustrating how ECM components, blood vessels, paracrine factors and innervation coordinately modulate satellite cell progression through quiescence, proliferation and differentiation. Quiescent Pax7⁺ MyoD⁻ cells are preserved via Notch, p57 and FoxO signaling, accompanied by FoxO3-mediated autophagy and elevated oxidative phosphorylation. Stimulation from the niche initiates proliferative Pax7⁺ MyoD⁺ status driven by HGF, YAP and STAT3; HGF/c-Met-PI3K-Akt and YAP/TAZ-TEAD cascades are activated alongside metabolic shift toward glycolysis. Downregulation of Pax7 and induction of MRF4/MyoG trigger terminal differentiation, where MyoD/Myf5-E-box axis drives myogenic gene transcription, p21 and PGC-1α facilitate cell-cycle exit, and oxidative phosphorylation is restored to support myofiber maturation

Identification and characterization of SCs rely on specific molecular markers (proteins, transcription factors, etc.) that label them during quiescence, activation, and differentiation (Fig. 1). The transcription factor Pax7 is the most critical and highly specific marker, playing a decisive role in maintaining quiescence, self-renewal, and myogenic differentiation. In the quiescent state, SCs rely on Pax7 to suppress premature differentiation-related genes (e.g., MyoD), ensuring retention in the G0 phase and stability of the stem cell pool.^21^ Upon muscle injury or external stimuli, the Notch signaling pathway is activated, and Pax7^+^ SCs enter the proliferation phase. Some cells downregulate Pax7 and upregulate MyoD, initiating myogenic differentiation.^22^ MyoD is a key marker of SC activation and commitment to differentiation, inducing cell cycle progression and proliferation while initiating downstream myogenic gene expression.^23^ The dynamic balance between Pax7 and MyoD expression jointly regulates SC self-renewal and differentiation decisions: high Pax7 expression favors self-renewal, while low Pax7 promotes terminal differentiation.

### Maintenance of satellite cell quiescence

Satellite cells typically exist in a quiescent state (QSC), characterized by cell cycle arrest in G0 phase and low metabolic activity reliant on oxidative phosphorylation. The Notch signaling pathway is key to maintaining quiescence. Ligand binding to Notch receptors triggers γ-secretase-mediated cleavage, releasing the Notch intracellular domain, which activates Hes/Hey family transcription factors that suppress myogenic differentiation, thereby sustaining cellular quiescence.^24^ Concurrently, high expression of cyclin-dependent kinase inhibitors such as p57Kip2 inhibits the Cyclin E-CDK2 complex, preventing G1/S transition and ensuring SC quiescence.^25^ Upon skeletal muscle injury or external stimuli, quiescent SCs rapidly activate by downregulating Notch signaling and activating Wnt and TGF-β superfamily pathways. Hepatocyte growth factor (HGF) released after injury binds c-Met receptors on SCs, promoting cell cycle re-entry via PI3K-Akt and MAPK pathways. Mechanical stress also activates SCs through integrin-mediated signaling and YAP/TAZ activation.^12^ Regulation of SC state transitions involves precise coordination of transcription factors: Pax7 is highly expressed in quiescent and early-activated cells and interacts with MyoD to regulate cell fate. MyoD expression marks entry into proliferation and initiation of myogenic differentiation, a process modulated by E proteins and Id proteins.^26^ Recent studies indicate that the FoxO transcription factor family helps maintain SC quiescence by regulating autophagy and antioxidant stress-related genes, enabling adaptation to metabolic and environmental challenges.^5^

### Regulations of satellite cell activation and proliferation

Activation of SCs in response to injury or stress is a highly coordinated, multi-step process integrating signals from damaged muscle fibers, the local microenvironment, and systemic circulation. Muscle injury disrupts sarcolemmal integrity, releasing intracellular components such as ATP and calcium ions into the extracellular space and triggering a local inflammatory response, collectively providing initial activation signals.^27^ Rapid release of cytokines and growth factors in the injured muscle microenvironment is critical. Hepatocyte growth factor (HGF), one of the earliest identified SC activators, is released from the ECM and activated within minutes of injury. Binding to its receptor c-Met activates ERK/MAPK and PI3K/AKT pathways, promoting SC exit from quiescence and cell cycle entry.^28^ Members of the TGF-β superfamily, particularly myostatin, regulate the activation threshold; myostatin levels increase post-injury and negatively regulate SC proliferation via Smad2/3 signaling, an effect counteracted by antagonists like follistatin, allowing precise control of activation.^29^ Inflammatory cells and their cytokines play dual roles: neutrophils arriving early post-injury release TNF-α, promoting SC cell cycle entry via NF-κB signaling.^30^ Subsequently infiltrating macrophages phagocytose necrotic debris and secrete proinflammatory cytokines like IL-6, which activates the JAK/STAT3 pathway via gp130, promoting SC proliferation and inhibiting premature differentiation.^31^ IFN-γ from activated T cells regulates activation by increasing MHC class I expression and enhancing SC responsiveness to growth factors.^32^ Mechanical stress signals are also essential: physical deformation from muscle use or injury is sensed by SCs via integrin-mediated mechanotransduction. β1-integrin interaction with ECM proteins (e.g., fibronectin, laminin) activates FAK signaling, promoting cell cycle progression.^33^ Nuclear translocation of Hippo pathway effectors YAP/TAZ is critical; in the nucleus, YAP/TAZ collaborate with TEAD to promote proliferation-related gene expression (e.g., CTGF, CYR61).^12^ Metabolic signaling modulates SC activation: local hypoxia stabilizes HIF-1α, promoting glycolysis-related gene expression to meet energy demands of proliferation.^34^ Elevated lactate levels promote SC proliferation via GPR81-dependent mechanisms, and ketone bodies like β-hydroxybutyrate influence gene expression by inhibiting histone deacetylases and inducing epigenetic modifications.

### Satellite cell differentiation and myoblast fusion

After proliferation, SCs enter a differentiation program governed by multi-level regulation. Differentiation initiation involves cell cycle exit mediated by upregulation of inhibitors such as p21 and pRb. p21 inhibits G1/S transition by suppressing cyclin E-CDK2 activity, while hypophosphorylated pRb binds E2F transcription factors, repressing proliferation genes and promoting myogenic transcription.^35^ The Myogenic Regulatory Factor (MRF) family centralizes differentiation regulation: MyoD and Myf5 control proliferation, while Myogenin and MRF4 are key differentiation regulators. Myogenin upregulation directly activates muscle-specific genes like myosin heavy chain (MyHC), creatine kinase (CK), and myosin light chain (MLC).^36^ MRF proteins form heterodimers with E-proteins, recognize E-box sequences (CANNTG) in target gene promoters, and recruit chromatin remodeling complexes (e.g., SWI/SNF) and histone acetyltransferases (e.g., p300/CBP), promoting chromatin relaxation and transcriptional activation.^37^ The balance between Notch and Wnt signaling crucially regulates the proliferation-to-differentiation transition: Notch signaling suppresses MyoD activity and differentiation via Hes/Hey factors, maintaining proliferation, while Wnt signaling (e.g., Wnt7a) promotes differentiation via Frizzled receptors. BMP signaling also participates, inhibiting differentiation through Smad1/5/8 phosphorylation; upregulation of BMP antagonists like Gremlin1 and Noggin promotes differentiation.^38^ MicroRNAs (miRNAs) fine-tune differentiation post-transcriptionally: miR-1 and miR-206 are upregulated during differentiation, promoting myocyte differentiation by suppressing inhibitors like HDAC4 and DNMT3a;^39^ miR-133 regulates the balance between differentiation and proliferation by suppressing WHSC2, an accessory factor of serum response factor (SRF). Epigenetic mechanisms provide additional control: muscle-specific gene loci undergo chromatin remodeling with increased activation marks (e.g., H3K4me3, H3K36me3) and decreased repression marks (e.g., H3K27me3).^40^ Nuclear-cytoplasmic shuttling of HDAC4 and HDAC5 also regulates differentiation; these deacetylases recruit repressor complexes in the nucleus to suppress premature differentiation but are expelled upon differentiation signals, releasing inhibition on myogenic genes.^41^ ECM remodeling is crucial: upregulation of MMP activity (e.g., MMP-2, MMP-9) degrades basement membrane components, facilitating myoblast fusion.^42^ Increased expression of adhesion molecules like M-cadherin and N-CAM mediates myoblast recognition and adhesion, foundational for cell fusion.^43^ Metabolic reprogramming accompanies differentiation: myoblasts shift from glycolysis to oxidative phosphorylation via mitochondrial biogenesis regulated by PGC-1α.^44^ mTORC1 signaling supports protein synthesis and myotube formation early in differentiation but gradually declines to prevent excessive growth.^45^

Precise regulation of the satellite cell niche is essential for maintaining quiescence, activation, and differentiation. This specialized three-dimensional space, composed of the extracellular matrix, vascular system, innervation, and paracrine factors, critically determines SC fate decisions through complex interactions. The ECM provides structural support and biochemical signaling: major components like collagens (types I and IV), laminin, fibronectin, and proteoglycans (e.g., perlecan) interact with SCs via integrin receptors (e.g., α7β1 integrin), transmitting mechanical signals and activating intracellular pathways (e.g., FAK-Src, PI3K-Akt) that regulate survival, proliferation, and differentiation.^46^ The ECM is not static; SCs perceive its mechanical properties (stiffness, elasticity) and topological structure (nanoscale fiber arrangement), influencing their behavior.^47^ After muscle injury, MMPs (e.g., MMP-2, MMP-9) are activated, degrading original ECM components and remodeling a temporary matrix that facilitates SC migration and proliferation.^48^ The vascular system is a pivotal hub for energy and material exchange, maintaining niche homeostasis: a dense capillary network supplies oxygen and nutrients while removing metabolic waste.^49^ Vascular endothelial cells secrete signaling factors like VEGF, HGF, and IGF-1, directly promoting SC activation and proliferation.^50,51^ During muscle regeneration, angiogenesis and SC activation are spatiotemporally coordinated. Neural innervation maintains muscle fiber contraction via neurotransmitters (e.g., acetylcholine) and neuropeptides (e.g., CGRP, substance P) that directly or indirectly influence SC activity.^52^ Schwann cells and satellite glial cells secrete neurotrophic factors (e.g., NGF, GDNF) and cytokines, regulating niche homeostasis, especially during injury repair.^53^

Paracrine signaling networks are primary intercellular communication forms within the niche, precisely regulating SC fate transitions. IL-6, released by activated SCs, immune cells (e.g., macrophages), and damaged muscle fibers, plays a crucial role in the initial injury response.^31^ Binding to IL-6R activates the gp130/JAK/STAT3 pathway; STAT3 phosphorylation and nuclear translocation upregulate genes like cyclin D1, driving SCs from G0 into the cell cycle and initiating proliferation.^54^ IL-6 signaling is context-dependent: timely and appropriate signaling promotes regeneration, whereas sustained excess may lead to fibrosis.^55^ IGF-1 is another multifunctional paracrine factor with anabolic and mitogenic effects. Binding to IGF-1R activates PI3K-Akt and Ras-MAPK pathways.^56^ Akt activation promotes protein synthesis, cell survival, and inhibits apoptosis and autophagy by suppressing FoxO transcription factors; it also downregulates cell cycle inhibitors like p27Kip1, promoting SC proliferation and differentiation.^57^ Muscle-specific knockout of IGF-1 receptor in mice causes severe regeneration defects, confirming IGF-1 signaling’s indispensable role in vivo.^58^ Other crucial paracrine factors include TGF-β superfamily members like myostatin, which inhibit SC activation and differentiation via Smad2/3 signaling, counteracted by antagonists like follistatin.^29^ PDGF-BB secreted by vascular endothelial cells promotes SC migration, guiding stem cells to injury sites.^59^ Extracellular vesicles (including exosomes) have recently gained attention as novel paracrine messengers, encapsulating and delivering bioactive molecules (proteins, mRNA, miRNA) for precise intercellular genetic exchange and regulation of recipient SC behavior.^60^ Table 1 summarizes the key niche elements that support satellite cell function and their core roles.

**Table 1:** Satellite cell niche: key elements and core functions

Niche component	Key elements	Core functions
Extracellular matrix (ECM)	Collagens, Laminin, Fibronectin, Integrins, MMPs	Provides structural and mechanical support; Transmits biochemical signals;^46^ Remodeled after injury to enable SC migration.^48^
Vascular system	Capillaries, Endothelial Cells(secrete VEGF, HGF, IGF-1)	Delivers oxygen and nutrients;^49^ Secretes growth factors to directly activate and proliferate SCs;^50^ Coordinated with regeneration.^51^
Paracrine signaling	IL-6: From SCs, immune cells. IGF-1: From endothelial cells, etc. Myostatin: Negative regulator. PDGF-BB: Promotes migration.	IL-6: Drives cell cycle entry (via STAT3).^54^ IGF-1: Promotes growth & survival (via PI3K/Akt).^56^ Myostatin: Inhibits activation (via Smads).^29^ PDGF-BB: Guides SCs to damage.^59^ Vesicles: Carry genetic instructions.^60^

### Satellite cell heterogeneity

Early studies mostly regarded satellite cells as a functionally homogeneous stem cell population. However, recent advances in single-cell transcriptomics and lineage tracing technologies have confirmed that skeletal muscle SCs exhibit prominent molecular and functional heterogeneity. SCs can be categorized into multiple subpopulations based on core transcription factor expression, metabolic profiles, and cell fate potential; moreover, the composition and function of these subpopulations undergo age-dependent remodeling, serving as one of the core mechanisms driving the decline of muscle regenerative capacity in aged individuals.^61,62^

Based on Pax7 expression levels, quiescent and early-activated SCs can be classified into two core functional subpopulations: Pax7-high (Pax7^Hi^) and Pax7-low (Pax7^Lo^). These two subpopulations exhibit significant differences in stemness maintenance, self-renewal capacity, and differentiation potential. Pax7^Hi^ cells are enriched in stem cell-associated genes and mitochondrial genes; they possess stronger stemness maintenance capacity and oxidative metabolic adaptability, as well as greater self-renewal capacity, and are responsible for maintaining the long-term homeostasis of the stem cell pool. Following injury, this subpopulation can generate new Pax7^Hi^ cells through asymmetric division to replenish the stem cell reservoir, while simultaneously producing Pax7^Lo^ cells that enter the differentiation program; their characteristic marker genes include Ptprb, Pvalb, Acta1, and Hbb-bt. Pax7^Lo^ cells highly express genes associated with myogenic differentiation and belong to the category of directed myogenic progenitor cells. They exhibit stronger proliferative activity and are more prone to exit the cell cycle and initiate terminal differentiation. These cells serve as the primary functional cells for post-injury myofiber repair, and their characteristic markers include Cdh15, Rcan2, Rps28, and Acta2.^63^

In addition to classifications based on core transcription factors, single-cell RNA sequencing has further revealed the multidimensional heterogeneity of SCs throughout their entire cycle of quiescence, activation, and differentiation. Quiescent SCs do not constitute a single cell population but can be further subdivided into multiple subpopulations with distinct transcriptional profiles: one subpopulation highly expresses the Notch signaling pathway and cell cycle inhibitory factors, remains in a state of deep quiescence, and is responsible for the long-term maintenance of the stem cell pool; another subpopulation exhibits a “primed quiescent state” state, with low expression of certain quiescence markers, responds more rapidly to injury signals, and can quickly enter the proliferative cycle.^64^ During the activation and proliferation phase, SCs also exhibit heterogeneity in their differentiation tendencies: some cells are more inclined to return to a quiescent state after self-renewal, while others are directed toward myogenic terminal differentiation. This difference in fate is jointly regulated by gradient expression of Wnt and Notch signaling pathways and epigenetic modifications.^65^

The heterogeneous homeostasis of satellite cells is not maintained entirely autonomously but is highly dependent on regulation by the metabolic microenvironment constituted by muscle fibers. Granulocyte colony-stimulating factor (G-CSF), secreted by muscle fibers, is a key microenvironmental factor for maintaining the Pax7^Hi^ subpopulation, and its transcription is regulated by MyoD in a metabolism-dependent manner. Myofiber-derived G-CSF establishes and maintains the Pax7^Hi^ SC subpopulation in adult and aged mice by promoting the asymmetric division of Pax7^Hi^ and Pax7 moderately expressing (Pax7^Mi^) SCs; notably, exercise-induced G-CSF can partially restore the loss of Pax7^Hi^ cells in aged mice.^66^ The metabolic characteristics of different muscle fiber types also influence SC heterogeneity: glycolytic fast-twitch myofibers are enriched with Pax7^Hi^ cells, whereas the muscle fiber type transition accompanying aging (from glycolytic to oxidative) can partially explain the reduction in the Pax7^Hi^ subpopulation during the aging process. Furthermore, SCs derived from different muscle fiber types exhibit inherent differences in proliferative capacity; SCs derived from slow-twitch soleus myofibers demonstrate greater proliferative potential, which is associated with differences in the activation of the FGFR signaling pathway.^67^

During the aging process, the heterogeneity of satellite cells undergoes a characteristic remodeling, which is one of the core cellular mechanisms underlying the decline in muscle regenerative capacity in the elderly. Both the number and function of the satellite cell (SC) pool decrease with aging, and this decline exhibits significant subpopulation-specificity, with the Pax7^Hi^ subpopulation showing preferential depletion. Studies have shown that the proportion of Pax7^Hi^ SCs in the tibialis anterior muscle of aged mice is significantly lower than that in young mice, accompanied by downregulation of stem cell-associated genes (such as CD34) and upregulation of differentiation-related genes (such as MyoD and MyoG).^66^ Studies of muscle regeneration in young, middle-aged, and elderly mice (approximately 5, 20, and 26 months old), using scRNA-seq and spatial transcriptomics, further revealed an enrichment of senescent-like MuSC subsets in aged muscle; these cells are in a critical transitional state with significantly impaired regenerative capacity. Single-cell transcriptomic analysis confirmed that age-related changes in the SC transcriptome primarily stem from dysregulation at the chromatin and transcriptional levels, rather than changes in RNA stability, and that these activated genomic regions are more sensitive to restorative regulation by microenvironmental factors.^68^

Aging also induces the emergence of various functionally abnormal SC subpopulations. In elderly individuals, there are inflammation-associated SC subpopulations characterized by sustained activation of the NF-κB signaling pathway, upregulation of pro-inflammatory factors, and impaired myogenic differentiation potential; in addition, some cells undergo epigenetic enhancer rewiring, leading to downregulation of myogenic identity-related genes and loss of stem cell identity, which further exacerbates regenerative dysfunction. Single-cell multi-omics studies have confirmed that the overall heterogeneity of aging SCs increases; in addition to functionally normal subpopulations, there are multiple abnormal subpopulations, including those exhibiting aging-like characteristics, a tendency toward fibrosis, and metabolic dysregulation. These abnormal subpopulations further disrupt microenvironmental homeostasis through paracrine effects, creating a vicious cycle.^69,70^ A human skeletal muscle aging multi-omics atlas has validated the conservation of these patterns: in aged muscle, the myogenic potential of SCs is universally reduced, and susceptibility subpopulations associated with sarcopenia emerge simultaneously. Changes in chromatin accessibility directly influence the expression of genes related to muscle regeneration, constituting a key cellular basis for the development of age-related sarcopenia.^71^

## Functional declines in satellite cells in aged skeletal muscle

### Phenotypic and functional alterations in aged satellite cells

**Fig. 2:**
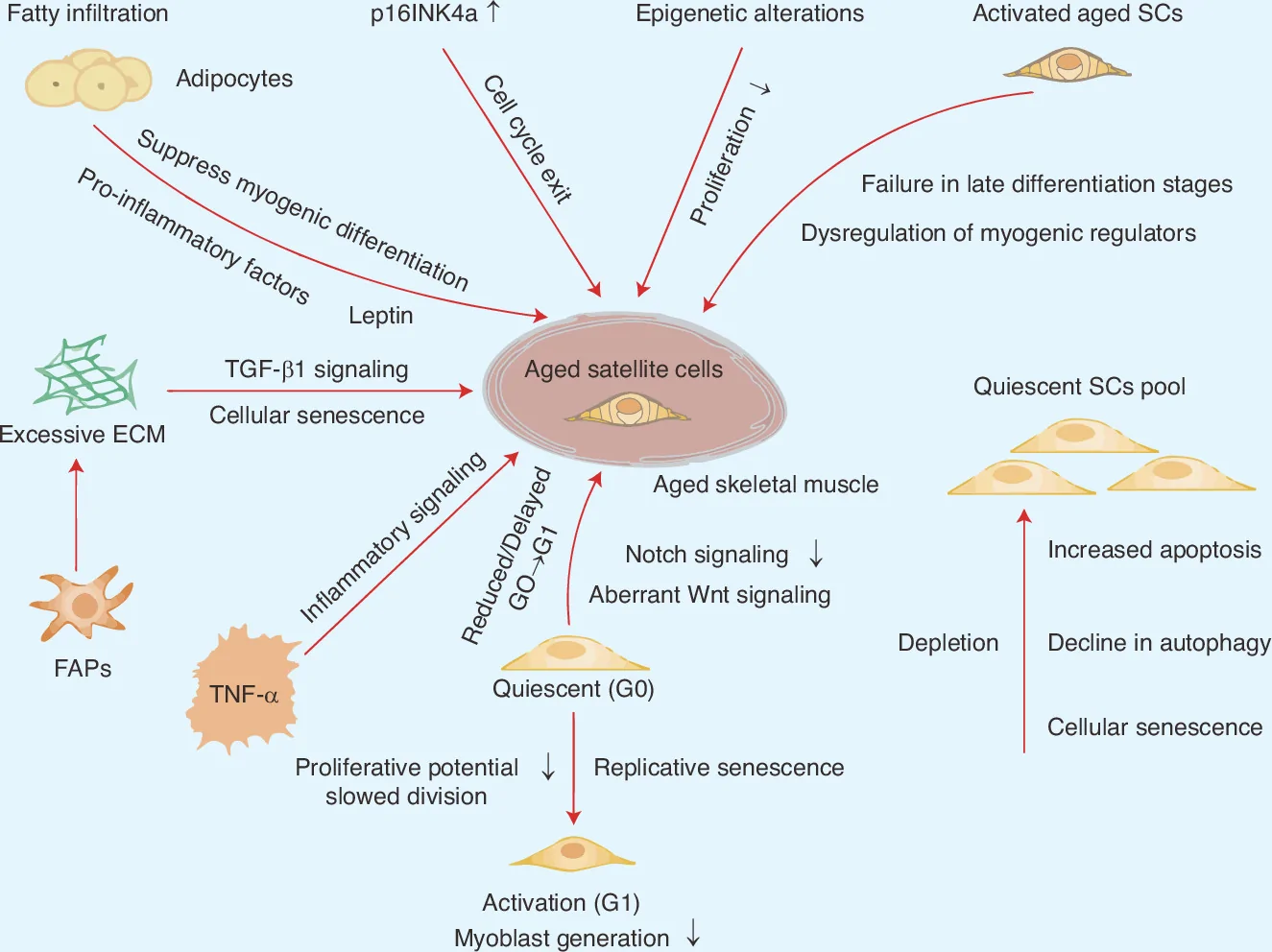
Satellite cells in aged skeletal muscle. Diagram summarizes cell-intrinsic and niche-derived alterations that impair satellite cell (SC) function during skeletal muscle aging. Extrinsic changes—adipocyte infiltration, fibro-adipogenic progenitor (FAP) activation, excessive ECM deposition, elevated TGF‑β1, pro-inflammatory cytokines, altered metabolic cues such as leptin—promote chronic inflammation, fibrosis, and paracrine suppression of myogenesis. Intrinsic defects include epigenetic remodeling, p16INK4a induction with cell-cycle exit, decreased Notch and aberrant Wnt signaling, reduced autophagy and altered proliferation. These events cause delayed G0–G1 transition, diminished proliferative/self‑renewal capacity, replicative senescence, increased apoptosis, and failure of late myogenic differentiation, ultimately reducing myoblast production and regenerative capacity, impairing tissue repair in aged skeletal muscle

The most prominent feature of aging skeletal muscle is progressive fiber atrophy, particularly selective loss of fast-twitch type II fibers, leading to declines in mass and strength. This atrophy is closely associated with reduced SC numbers and functional decline. In aging, the SC pool gradually depletes, and remaining cells undergo intrinsic alterations: they become less responsive to activation signals, slow to transition from quiescence (G0) to cell cycle (G1), have reduced proliferative capacity, and are more prone to senescence or apoptosis.^72^ This functional decline correlates with accumulated age-related epigenetic changes, such as altered DNA methylation and histone modifications, suppressing pro-proliferative and myogenic genes.^73^ Aged SCs overexpress the cyclin-dependent kinase inhibitor p16INK4a, a senescence hallmark causing permanent cell cycle arrest.^2^ Regenerative capacity decline is also manifested as reduced differentiation efficiency: even when aged SCs proliferate, they often fail to fuse effectively into new myofibers or repair damaged ones, associated with dysregulation of myogenic regulators like MyoD and Myogenin. With aging, skeletal muscles undergo structural remodeling characterized by fibrosis from adipose tissue infiltration and excessive ECM deposition, reinforcing muscle fiber loss and functional decline. Fat infiltration (muscular adiposis) involves abnormal adipocyte accumulation between epimysium and endomysium, originating from fibro-adipogenic progenitor (FAP) cells rather than SCs. In young muscle, FAPs transiently proliferate post-injury, support SC myogenesis via cytokine secretion, and are eliminated by apoptosis, maintaining population stability. In aging, chronic low-grade inflammation drives FAP adipogenic differentiation: elevated proinflammatory cytokines (e.g., TNF-α) and altered BMP and Wnt signaling misdirect FAP differentiation toward adipocytes instead of regenerative support.^74^ Fibrosis involves excessive deposition of ECM components like collagen. Under normal conditions, transient ECM deposition provides a scaffold for regeneration and transmits mechanical and biochemical signals; however, during the aging process, the sustained, excessive activation of the fibrogenic TGF-β1 pathway stimulates fibroblasts and FAPs to synthesize large amounts of collagen I and III and other ECM proteins, resulting in the formation of dense, inelastic fibrous scar tissue. This fibrotic environment physically impedes SC migration and new myofiber alignment and suppresses SC function via signaling, creating a vicious cycle obstructing regeneration.^75^ Adipose infiltration and fibrosis collectively constitute a dysfunctional microenvironment: adipocytes secrete proinflammatory factors and leptin, exacerbating local inflammation and suppressing myogenic differentiation, while fibrotic tissue increases stiffness and alters mechanical signaling. Abnormal ECM also isolates key growth factors, reducing bioavailability. These alterations severely compromise regenerative potential, rendering residual SCs functionally unsustainable (Fig. 2).

### Attrition of the quiescent satellite cell pool

Maintaining the SC pool requires the long-term survival and self-renewal of quiescent cells; however, with advancing age, this pool undergoes a dual change characterized by progressive depletion in number and a comprehensive decline in the function of the remaining cells, which together drive the decline in skeletal muscle regenerative capacity in older adults. Contributing factors include persistent apoptosis increased by pro-inflammatory signals and diminished survival factor support in the aging microenvironment. More critically, abnormal activation of cellular senescence plays a key role: senescent SCs significantly increase p16INK4a expression, causing irreversible cell cycle exit. These “zombie cells” survive but lose proliferative and differentiation capacity. Age-related decline in autophagy impairs clearance of damaged components and homeostasis maintenance, accelerating SC attrition.^5^

The theory of “cellular survivorship bias,” proposed in recent studies, offers a novel mechanistic perspective on the remodeling of the quiescent SC pool during aging. During aging, the SC pool does not undergo uniform functional decline; rather, adaptive selection occurs at the cellular level. The tumor suppressor NDRG1 is significantly upregulated in aged SCs and modulates the proliferative cell phenotype by inhibiting the PI3K-AKT-mTOR signaling pathway. On the one hand, NDRG1 enhances the stress-resistant survival capacity of stem cells, helping them persist long-term in the harsh microenvironment of aging tissues; on the other hand, this molecule inhibits the rapid activation process of stem cells, thereby reducing the efficiency of immediate regeneration following injury. Although SCs with low NDRG1 expression possess greater activation and regenerative potential, they struggle to adapt to the chronic stress microenvironment of aging tissues and are gradually cleared as age advances. SCs with high NDRG1 expression are selected and retained in the quiescent pool due to their greater stress resistance; however, because mTOR signaling is continuously suppressed, they are unable to enter the mTOR-dependent GAlert pre-activation state. This directly leads to a delay in the transition from the quiescent state (G0) to the cell cycle (G1), resulting in a slow response to activation signals. Although artificially knocking out NDRG1 in aged SCs can restore the cells’ rapid activation capacity and enhance regeneration efficiency following a single injury, it significantly impairs SC survival. Under repeated injury stimuli, the SC pool depletes more rapidly, which in turn leads to a severe decline in subsequent regenerative capacity. If NDRG1 is knocked out in SCs during the young stage, the size of the SC pool in mice upon reaching old age will be significantly smaller than that of age-matched wild-type mice, and muscle regeneration defects will be more severe, further confirming that the NDRG1-mediated survival bias is a compensatory mechanism for maintaining the longevity of the aged SC pool.^76^

Reduction in the quiescent pool directly limits stem cells available for mobilization and regeneration post-injury. Even remaining SCs exhibit comprehensive functional impairment: activation capacity is significantly reduced, with slow response to activation signals. Notch signaling, crucial for maintaining quiescence and promoting activation, becomes less active with age, while abnormal Wnt activation disrupts equilibrium, hindering effective activation.^3^ Transition from quiescence (G0) to cell cycle (G1) is delayed, linked to altered cell cycle regulator function. When activated, senescent SCs have limited proliferative potential, resulting in inefficient expansion and diminished growth capacity, likely due to replicative senescence mechanisms like telomere shortening and sustained DNA damage responses.^2^ Cell division slows, leading to premature cell cycle exit and insufficient myoblast generation for repair. This proliferative defect correlates with metabolic reprogramming dysfunctions, such as diminished mitochondrial function unable to meet energy and biosynthetic demands of rapid proliferation. Ultimately, differentiation ability is compromised: aged SCs often fail in late differentiation stages, unable to properly express MyoD and Myogenin, leading to inefficient myotube formation.^77^ Differentiation outcomes may deviate from normal myogenesis, making cells prone to apoptosis or atypical characteristics. This functional decline is closely associated with epigenetic alterations accumulated with aging, such as increased inhibitory chromatin marks (e.g., H3K27me3) at key myogenic gene loci, impeding normal gene expression and disrupting myogenic transcriptional programs.^73^

It is worth noting that the NDRG1-mediated survival bias differs fundamentally from the classical antagonistic polygenic theory of aging. The antagonistic polygenic hypothesis posits that certain genes exhibit bidirectional effects: when expressed during an organism’s youth, these genes enhance an individual’s fitness for survival and reproduction, conferring an evolutionary advantage. However, as an individual enters old age, the continued expression of the same genes induces tissue functional decline and produces negative physiological effects.^78^ Natural selection tends to favor the retention of genetic traits that are advantageous in early life, while the negative effects that arise in old age are selectively ignored. In contrast, high expression of NDRG1 does not confer any benefits to young stem cells; rather, it directly inhibits the regenerative response following muscle injury. Only within the harsh microenvironment of aged tissues does NDRG1 confer enhanced stress-resistant survival capacity to stem cells, leading to the selective retention of cell subpopulations with high NDRG1 expression. This molecular characteristic is the result of selection for long-term cellular survival, rather than a passive byproduct of gene antagonistic pleiotropy. This conclusion also suggests that age-related changes in gene expression cannot be uniformly regarded as negative consequences of cellular damage; rather, some molecular remodeling represents compensatory regulation by cells to maintain the stem cell reservoir and prevent the complete loss of tissue repair capacity.

## Molecular mechanisms of satellite cell senescence

### Telomere shortening and replicative senescence

**Fig. 3:**
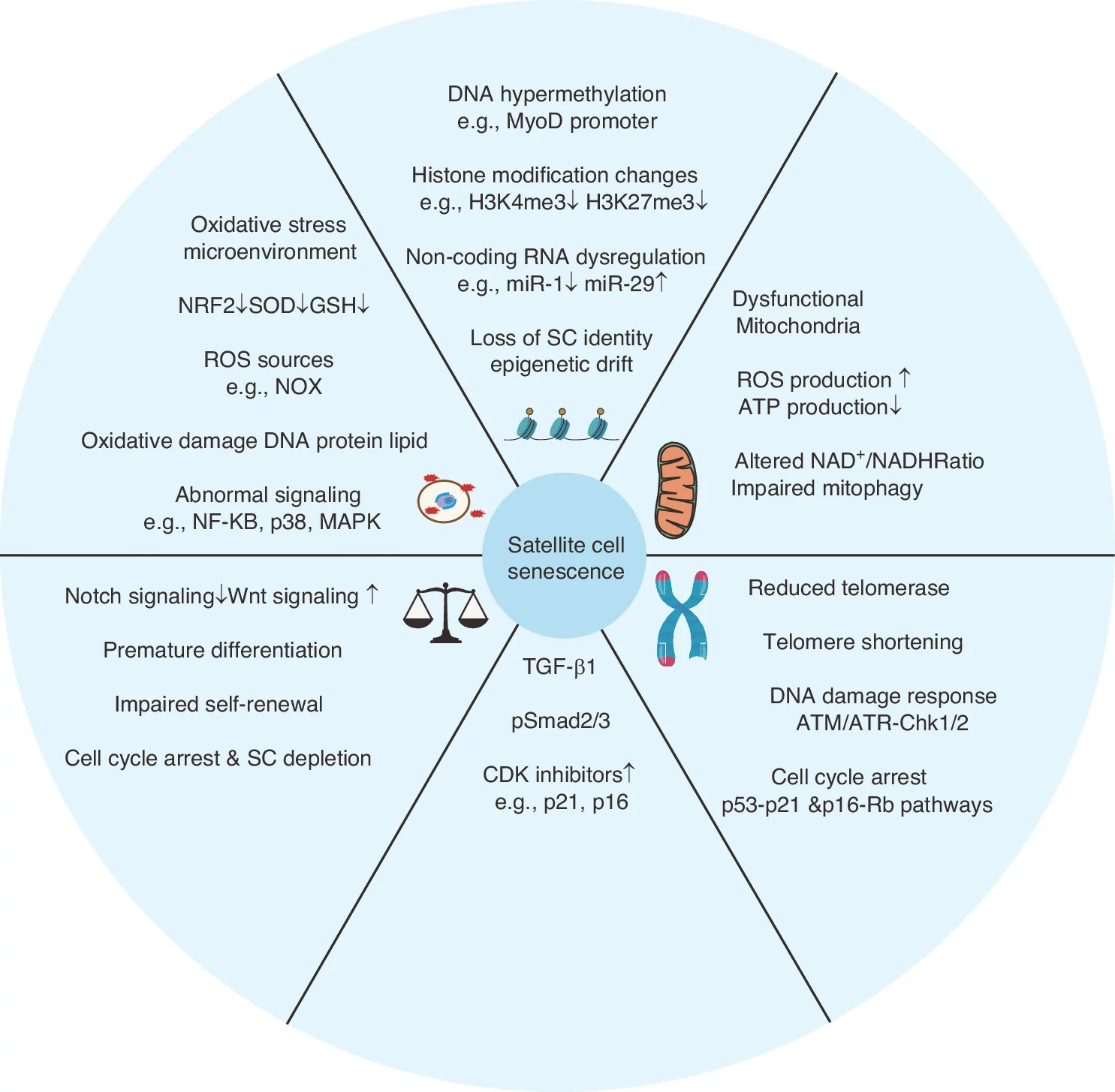
Molecular mechanisms of satellite cell senescence. Peripheral segments illustrate contributing processes: epigenetic alterations (DNA hypermethylation, histone modification, noncoding RNA dysregulation) that erode stem cell identity; mitochondrial dysfunction with increased ROS, decreased ATP and impaired mitophagy; telomere shortening and reduced telomerase triggering DNA damage responses and p53/p21 or p16/Rb-mediated arrest; elevated TGF-β1/pSmad signaling raising CDK inhibitors; disrupted Notch/Wnt balance causing premature differentiation and loss of self-renewal; and oxidative stress and inflammatory signaling (NF-κB, p38 MAPK) promoting oxidative damage. Collectively, these pathways culminate in cell cycle arrest and satellite cell attrition. This network thereby impairs muscle regeneration with aging

Telomeres, protective chromosome end structures, progressively shorten with each cell division, ultimately triggering replicative senescence. In SCs, excessive telomere shortening induces irreversible cell cycle arrest via p53-p21 and p16INK4a-Rb pathways. Telomere attrition directly causes SCs to exit the cell cycle and triggers sustained DNA damage response, activating the ATM/ATR-Chk1/2 cascade and intensifying senescence.^72^ Telomere shortening and oxidative stress interact closely: reactive oxygen species (ROS) accelerate telomere shortening, and shortened telomeres are more susceptible to oxidative damage, forming a positive feedback loop accelerating aging (Fig. 3). In senescent SCs, telomerase activity is usually significantly reduced, impairing telomere repair and resistance to replicative senescence.^2^ Telomere dysfunction also affects SC epigenetic state: telomere shortening can alter heterochromatin structure, affecting telomere-proximal gene expression and disrupting normal cellular function, potentially further accelerating SC aging in a vicious cycle.^79^

### Mitochondrial dysfunction and metabolic dysregulation

Mitochondria, as the centers of cellular energy metabolism, play a key role in skeletal muscle satellite cell depletion when their function is disrupted. Aged SCs exhibit pronounced mitochondrial dysfunction, primarily abnormal network architecture and reduced functional efficiency, accompanied by decreased oxidative phosphorylation efficiency, reduced ATP production, and increased ROS generation.^5^ Even more seriously, the efficiency of mitochondrial autophagy also declines with age, making it unable to clear damaged mitochondria in a timely manner. This leads to the accumulation of dysfunctional organelles, which further exacerbates mitochondrial damage. Impaired metabolic reprogramming is another critical factor: aged SCs fail to efficiently transition metabolic modes from quiescence to activation, severely constraining proliferation capacity.^80^ Mitochondrial dysfunction leads to the massive accumulation of ROS. This process plays an upstream regulatory role: it not only induces oxidative stress and causes DNA damage but also disrupts epigenetic modification patterns while accelerating telomere shortening. Together, these factors gradually establish a positive feedback loop of aging driven by mitochondrial damage, ROS accumulation, and progressive telomere shortening. In addition to disrupting the cellular energy supply system, various metabolic stresses can also alter the NAD^+^ /NADH ratio, levels of key metabolic molecules such as α-ketoglutarate and S-adenosylmethionine. This, in turn, regulates the catalytic activity of epigenetic modification enzymes—such as deacetylases and DNA methyltransferases—thereby forming a molecular network in which metabolic status, oxidative stress, and epigenetic regulation are intricately intertwined, ultimately leading to the complete disruption of the satellite cells’ own physiological homeostasis.^81^

### Epigenetic dysregulation

Epigenetic regulation is crucial for maintaining SC homeostasis and function; its age-related dysregulation is a core mechanism driving SC attrition. This regulatory dysregulation is not an isolated abnormality in gene expression, but rather a downstream amplification mechanism jointly mediated by mitochondrial metabolic disorders, ROS-induced oxidative damage, and telomere dysfunction. It primarily manifests as abnormalities in DNA methylation patterns, disruptions in histone modifications, and imbalances in non-coding RNA expression; these factors collectively lead to the gradual loss of stem cell identity and functional decline.

Studies reveal a genome-wide trend toward hypomethylation with aging, particularly in gene deserts and repetitive sequences, potentially increasing genomic instability. In contrast, specific gene promoter regions exhibit abnormal hypermethylation, especially those critical for SC function. Methylation remodeling at the p16INK4a gene locus is one of the most characteristic epigenetic markers of aging satellite cells. In young cells, the p16INK4a promoter is highly methylated and transcriptionally silenced; during the aging process, this region undergoes specific demethylation, leading to high levels of p16INK4a expression, which induces G1 phase arrest by inhibiting pRb phosphorylation.^82^ In addition to p16INK4a, age-dependent methylation remodeling occurs widely in cell cycle regulatory genes: the promoters of inhibitory genes such as p21 and p27 undergo demethylation, while the promoters of proliferative genes such as cyclin D1 and CDK4 become highly methylated, completely disrupting the cell cycle. Abnormal methylation of myogenic regulatory genes is equally significant: high methylation of CpG islands in the MyoD and myogenin promoters directly silences the myogenesis program, while dysregulation of Pax7 methylation disrupts satellite cell self-renewal, leading to the continuous depletion of the quiescent stem cell pool.^73^ The aforementioned whole-genome and site-specific methylation imbalances are jointly regulated by mitochondrial metabolic dysregulation and the accumulation of intracellular reactive oxygen species. Mitochondrial defects lead to an insufficient supply of the methyl donor S-adenosylmethionine, while simultaneously depleting α-ketoglutarate, a key cofactor for demethylation, thereby causing the simultaneous inactivation of methyltransferases and demethylases.^83^ Accumulated intracellular reactive oxygen species can oxidatively modify DNA bases, interfering with the recognition of genomic targets by methyltransferases, and ultimately leading to a characteristic modification pattern characterized by genome-wide low methylation and high methylation at the promoters of functional genes.^84^

Histone modification states alter: H3K27me3, a crucial repressive mark, shows significant genomic distribution changes with age. In young SCs, H3K27me3 precisely marks developmental regulatory and lineage-nonspecific genes to maintain differentiation, but in aged cells, this pattern disrupts, potentially abnormally activating or suppressing genes. Enrichment of activating marks (e.g., H3K4me3, H3K36me3) in myogenic gene promoters decreases, further weakening myogenic expression. Histone acetylation balance disrupts with dysregulated HDAC and HAT activity, leading to chromatin compaction and gene suppression.^85^ Disruptions in histone modifications do not occur in isolation; cellular metabolic levels directly determine the catalytic activity of various modifying enzymes. Mitochondrial damage leads to a sharp decline in intracellular NAD^+^ levels. As NAD^+^ serves as a key coenzyme for deacetylases, its deficiency directly downregulates the function of the sirtuin family of proteins. α-Ketoglutarate is an essential substrate for histone demethylases; mitochondrial metabolic dysfunction leads to its depletion, persistently disrupting genomic methylation patterns, thereby establishing a pathway through which metabolic abnormalities drive epigenetic dysregulation. At the same time, the massive accumulation of reactive oxygen species can oxidatively damage various histone modification enzymes, disrupting the normal distribution of epigenetic marks such as H3K27me3 and H3K4me3. Combined with the reduced deacetylase activity caused by NAD^+^ depletion, these multiple factors collectively induce a comprehensive imbalance in histone modifications, continuously accelerating the aging process of satellite cells.^86,87^

Non-coding RNA network dysregulation, such as altered microRNA expression, promotes cell cycle progression and myogenic differentiation by downregulating miRNAs like miR-1 and miR-206, while inhibiting miRNAs like miR-29 and miR-34 families are upregulated. The miR-29 family targets cell cycle regulators and ECM genes, suppressing SC proliferation and promoting fibrosis. Altered long non-coding RNA (lncRNA) expression also plays regulatory roles; e.g., some lncRNAs recruit chromatin-modifying complexes to specific genomic sites, influencing gene expression. Various types of non-coding RNAs form intricate regulatory networks through cross-linking with one another and act in concert with epigenetic enzymes to collectively determine the differentiation pathway of myogenic cells.^86^ Aging triggers a global remodeling of the non-coding RNA network; disruption of this post-transcriptional regulatory system is a key pathway driving endogenous functional aging in satellite cells. Upon upregulation, miR-29 and miR-34 specifically target and inhibit positive regulatory molecules such as DNMT3A, B-Myb, and CDK6, inducing methylation dysregulation and blocking cell proliferation; miR-34 also accelerates aging by silencing SIRT1.^87^ Decreased expression of miR-1 and miR-489, which maintain stem cell homeostasis, leads to differentiation dysregulation and premature activation of differentiation, respectively.^88^ Imbalances in the expression of long non-coding RNAs such as H19 and MEG3, as well as circular RNAs, can recruit inhibitory modification complexes to enrich H3K27me3 and silence stemness genes. CircRNA dysregulation disrupts the endogenous ceRNA competitive regulation network, further amplifying the aging phenotype.^89,90^ Various types of non-coding RNAs synergistically regulate stem cell aging and provide numerous molecular targets for targeted interventions.^91^ Intracellular oxidative stress induced by mitochondrial dysfunction can regulate non-coding RNA transcription; in turn, dysregulated non-coding RNAs inhibit the mitochondrial autophagy pathway, promoting the continuous accumulation of reactive oxygen species and forming an intracellular negative feedback loop that continuously disrupts epigenetic homeostasis.

Abnormal epigenetic patterns can be inherited during cell division, progressively amplified as epigenetic drift, causing SCs to gradually deviate from normal epigenetic state, blur identity markers, and sustain functional decline. Epigenetic alterations closely interact with other aging drivers: metabolic dysregulation can modulate epigenetic modifier enzyme activity by affecting metabolites like S-adenosylmethionine and α-ketoglutarate, while DNA damage and telomere shortening can trigger specific epigenetic responses.^92^ At the same time, sustained oxidative stress not only directly damages DNA and proteins but also promotes the phosphorylation of chromatin remodeling complexes by activating stress signaling pathways such as p38 MAPK, thereby altering their genomic localization and activity. This integrates metabolic stress, oxidative damage, and the epigenetic programming of cell fate into a unified regulatory network at multiple levels.^93^

### Key signaling pathway dysregulation

The disruption of various signaling pathways during the aging process is not caused by abnormalities in a single pathway, but rather represents the final outcome resulting from the combined effects of multiple factors, including mitochondrial metabolic dysfunction, the accumulation of reactive oxygen species, and epigenetic dysfunction. In this process, the balance between the Notch and Wnt signaling pathways is disrupted. Coupled with the persistent, excessive activation of TGF-β and its downstream Smad signaling pathway—which can aggregate and integrate various upstream aging-related signals—this constitutes the core molecular event leading to the continuous depletion of the satellite cell reservoir.^75^ Notch and Wnt pathways play antagonistic yet synergistic roles in regulating SC fate; maintaining their equilibrium is crucial for homeostasis. In young muscle, Notch signaling primarily promotes SC proliferation and stemness maintenance. Post-injury, Notch activation prompts SC transition from quiescence to proliferation and expansion.^24^ As regeneration progresses, Notch activity gradually diminishes while canonical Wnt pathway upregulation drives proliferating SCs toward differentiation into myoblasts, ultimately fusing into new fibers or repairing damaged ones.^3^ This temporal regulation ensures orderly regeneration. However, during the aging process, this delicate balance is disrupted. The key changes are manifested as a significant reduction in Notch signaling activity and non-physiological, abnormal activation of the Wnt pathway; the accumulation of upstream oxidative stress and epigenetic dysregulation jointly mediate this imbalance in the pathway. Excessive reactive oxygen species can directly downregulate the expression of the Notch ligand Delta, disrupting the signaling function of the RBP-J molecule; aging-induced hypermethylation of genes also silences key coding genes of the Notch pathway, which, combined with abnormal metabolic signals released by mitochondrial metabolic dysfunction, leads to sustained, excessive activation of the canonical Wnt pathway. Within the aging skeletal muscle microenvironment, the synthesis of Notch ligands such as Delta continues to decline, and the function of key signaling molecules such as RBP-J is impaired, directly leading to the disruption of Notch signaling. Satellite cells struggle to exit the quiescent state and initiate proliferation, and the self-renewal capacity of stem cells declines simultaneously.^94^ Meanwhile, Wnt signaling inappropriately activates with age; though systemic Wnt levels may not elevate in aged mouse serum, local Wnt activity (particularly canonical pathway) increases in skeletal muscle microenvironment. This abnormal Wnt activation drives SCs toward premature differentiation, depleting the diminished quiescent pool and potentially propelling SCs toward non-myogenic senescence or apoptosis.^95^ Notch/Wnt imbalance collectively leads to declined SC regenerative potential: diminished Notch prevents effective proliferation initiation, while abnormal Wnt directs fate toward abnormal differentiation or attrition, ultimately impairing aged muscle repair capacity.^96^

The TGF-β signaling pathway, particularly Smad-mediated canonical pathway, plays a key role in suppressing SC function during aging.^97^ In young individuals, TGF-β signaling is kept low and works with other factors to maintain homeostasis, but in aging, TGF-β1 levels significantly elevate in circulation and local muscle tissues. Studies have found that TGF-β1 concentrations in the serum of elderly humans and aged mice are abnormally elevated, with sources including platelets, immune cells, and muscle parenchymal cells, among others. This excessive activation is the synergistic result of oxidative stress, inflammatory responses, and epigenetic dysregulation. In particular, the accumulation of ROS strongly induces TGF-β1 secretion by cells in the local microenvironment, while the epigenetic activation of aging-related genes such as p16 and p21 further amplifies Smad signaling.^98^ Hyperactivated TGF-β1 binding to its receptor induces phosphorylation of Smad2/3 (pSmad3), forming complexes that translocate into the nucleus and upregulate CDK inhibitors (e.g., p15, p21, p27, p16), forcing SCs to exit cell cycle, inhibiting proliferation, and inducing senescence.^99^ Direct antagonism exists between TGF-β/pSmad3 and Notch signaling: Notch activation blocks TGF-β-dependent CDK inhibitor upregulation, while Notch inhibition induces their expression. Molecularly, Notch activity governs pSmad3 binding to CDK inhibitor gene promoters. Thus, in aged muscle, declined Notch and elevated TGF-β/pSmad3 signaling exacerbate SC cell cycle arrest.^100^ TGF-β1 inhibition in aging extends beyond endocrine mechanisms; paracrine and autocrine pathways play significant roles. The aging muscle microenvironment itself (muscle fibers, immune cells, ECM) may continuously produce TGF-β1, creating an inhibitory local environment suppressing resident SC function.^101^ Targeting TGF-β signaling could therapeutically improve age-related muscle regenerative impairment: in animal models, systemic administration of TGF-β receptor kinase inhibitors reducing TGF-β signaling in skeletal muscle successfully enhanced aged mouse muscle regenerative capacity, suggesting TGF-β/Smad modulation as an effective treatment for SC attrition and muscle aging.^102^

It is important to note that Wnt and TGF-β signaling do not always play a singular “harmful” role in muscle regeneration and aging; their functions are highly context-dependent, and simply characterizing them as “age-promoting” or “regeneration-promoting” signals does not reflect their complex biological nature. Regarding the Wnt pathway, although age-related abnormal activation of Wnt drives premature differentiation and fibrosis of satellite cells,^103^ studies have also shown that Wnt signaling is indispensable during specific stages of muscle regeneration.^3,104^ Wnt7a drives the symmetric expansion of satellite stem cells by activating the planar cell polarity pathway, thereby expanding the stem cell pool,^95^ while Wnt3a acts in concert with Notch signaling to promote satellite cell proliferation in the early stages of injury; conversely, the Wnt/β-catenin pathway actively initiates the myogenic differentiation program during the differentiation stage.^105^ Therefore, the impact of Wnt signaling on satellite cell fate is highly dependent on signal intensity, timing, and cellular context; physiological and transient Wnt activation is essential for regeneration, whereas persistent, excessive, and abnormal activation is pathologically significant. The key change in aging is not the “presence” or “absence” of Wnt signaling, but rather the disruption of its spatiotemporal dynamics—that is, a shift from physiological, phasic activation to persistent, abnormal elevation. Similarly, TGF-β signaling also exhibits duality. Although this review emphasizes that excessive TGF-β/Smad activation during aging promotes cell cycle exit and fibrosis,^106^ TGF-β also performs important physiological functions during the early stages of muscle regeneration. It maintains the proliferative reserve of satellite cells by inhibiting premature differentiation, regulates the timely resolution of inflammatory responses, and promotes moderate remodeling of the extracellular matrix to provide a regenerative scaffold.^107^ Furthermore, there are functional differences among the various TGF-β subtypes: TGF-β1 primarily mediates fibrosis, while TGF-β2 and TGF-β3 play distinct roles in development and regeneration.^108^ How these differences change during the aging process remains to be thoroughly investigated. Therefore, pathological changes in TGF-β signaling during aging are essentially an imbalance in signaling homeostasis rather than an absolute increase in a single pathway; its beneficial effects in the early stages of regeneration and its harmful effects in the aging microenvironment depend on the duration and intensity of the signal, as well as its cross-regulation with other pathways. The core of pathological changes in aging-related signaling pathways lies in the disruption of signaling kinetics (intensity, duration, and spatiotemporal patterns), rather than in the unidirectional activation or inhibition of the pathways.

### Chronic inflammation and oxidative stress

ROS accumulation combined with declined natural antioxidant defense function creates an oxidative stress microenvironment around senescent SCs, a key mechanism causing functional exhaustion. As a central intermediary linking mitochondrial dysfunction, epigenetic abnormalities, signaling pathway imbalances, and telomere damage, this microenvironment permeates the entire process of satellite cell senescence and serves as a key hub mechanism driving their functional exhaustion. Normally, ROS act as vital signaling molecules regulating proliferation, differentiation, and apoptosis, but aging disrupts ROS production/clearance balance, leading to oxidative stress.^109^ Mitochondria are primary intracellular ROS sources; their dysfunction centralizes age-related oxidative stress. Senescent SC mitochondria exhibit electron transport chain defects, increasing electron leakage and excessive superoxide anion production. Age-related decline in mitophagy impairs timely clearance of damaged mitochondria, which persistently generate ROS, exacerbating oxidative damage.^110^ In addition to mitochondrial origins, the abnormal activation of enzymes in the NADPH oxidase (NOX) family is another significant source of ROS, particularly during inflammation. When facing a ROS threat, the antioxidant defence system of senescent skin cells (SC) is compromised: the activity of the core antioxidant transcription factor NRF2 decreases with age, reducing the expression of downstream antioxidant enzymes such as superoxide dismutase (SOD), catalase (CAT) and glutathione peroxidase (GPx).^111^ Simultaneously, biosynthesis of vital antioxidants like glutathione (GSH) decreases, while oxidized glutathione (GSSG) proportion increases, reducing cellular reducing capacity. It is important to note that oxidative stress cannot exert biological effects on its own; this physiological abnormality is intertwined with mitochondrial dysfunction and epigenetic dysregulation to form an integrated aging regulatory system.^112^ Structural and functional abnormalities in the mitochondrial electron transport chain are the primary drivers of increased intracellular reactive oxygen species (ROS) production. Excessively accumulated ROS can act on guanine-rich regions of the genome, interfering with the binding efficiency of DNA methyltransferases to chromatin targets, ultimately leading to pathological changes in the methylation patterns of gene promoter regions. Oxidative stress can also activate the p38 MAPK and NF-κB pathways; the activated kinases can directly mediate phosphorylation modifications of histones, simultaneously regulating the functional state of chromatin remodeling complexes and comprehensively reshaping the cell’s overall epigenetic landscape.^113^ Persistent gene silencing at the epigenetic level downregulates the transcription of core antioxidant transcription factors such as NRF2,^114^ and the synthesis of various antioxidant enzymes, such as superoxide dismutase and catalase, is also inhibited. The sustained decline in the cell’s overall capacity to clear reactive oxygen species further exacerbates mitochondrial structural and functional damage, thereby establishing a self-perpetuating feedback loop of aging. Persistent oxidative stress can also directly attack DNA sequences within telomere structures, accelerating the loss of telomere segments and thereby inducing replicative aging. ROS-mediated oxidative modifications can act on various proteins involved in epigenetic regulation, disrupting genomic DNA methylation processes, histone modification patterns, and regulatory networks composed of non-coding RNAs. At the signaling level, oxidative stress can downregulate the activity of the Notch pathway—which maintains stem cell pluripotency—and promote the pathological overactivation of the Wnt pathway (which mediates cell differentiation) and the TGF-β pathway (which induces cellular senescence). Inflammatory response programs dominated by the NF-κB and p38 MAPK pathways are also simultaneously triggered. These three pathological processes—oxidative stress, inflammatory responses, and mitochondrial damage—interact synergistically to progressively amplify the damaging effects associated with aging.^115^ Oxidative stress adversely affects multiple SC functions: elevated ROS induce oxidative DNA damage (e.g., 8-OHdG formation), activating DNA damage response pathways and causing cell cycle arrest. Oxidative protein modifications alter enzyme activity and signaling functions, while lipid peroxidation compromises membrane integrity.^93^ These multiple injuries ultimately lead to the loss of satellite cell stemness, the failure of their proliferative and differentiative capabilities, and the decline in muscle regeneration function.

## The aged niche and its impact on satellite cells

### Impact of the SASP

**Fig. 4:**
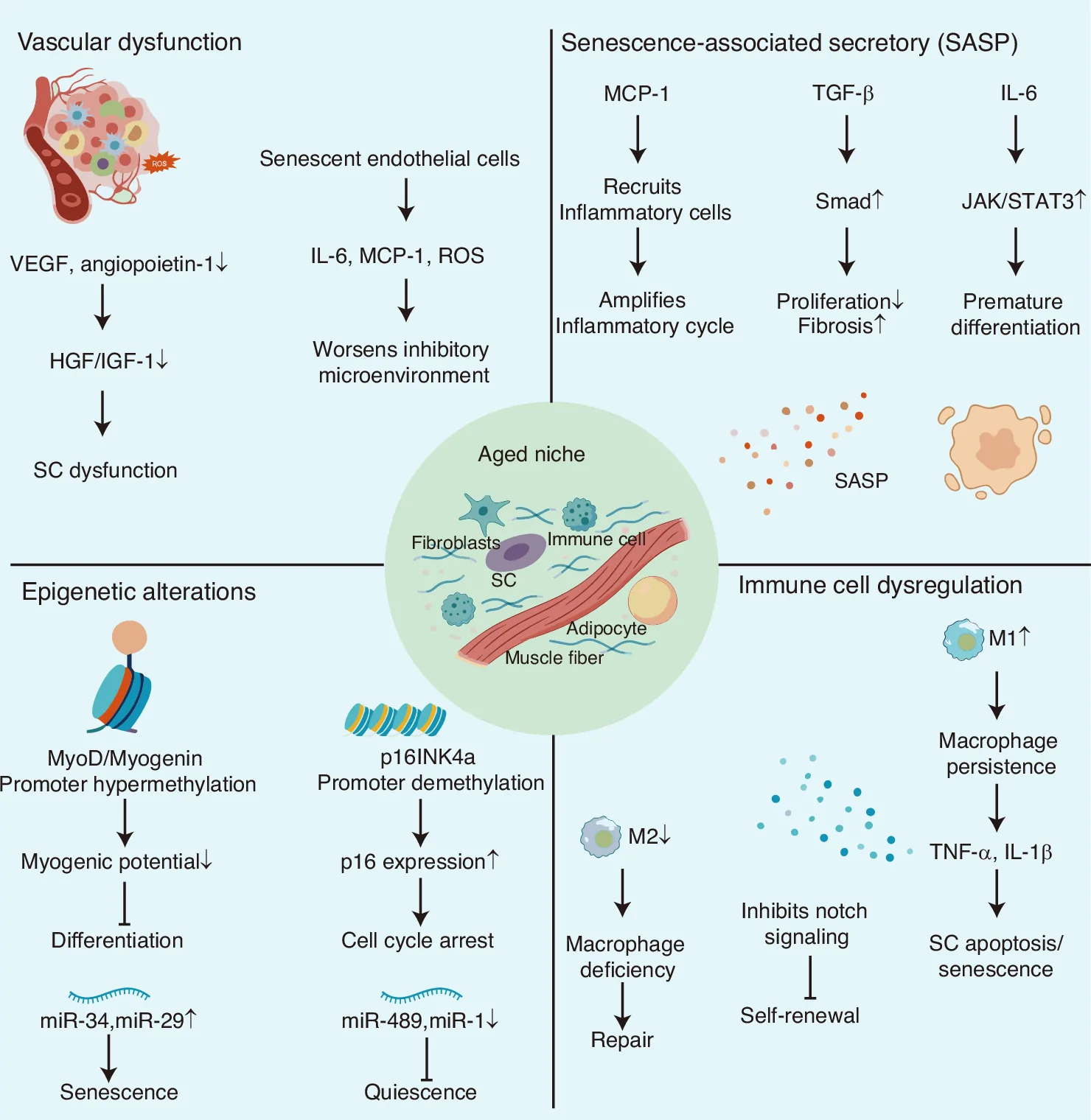
The niche driving satellite cell senescence. Central schematic depicts the aged muscle stem cell niche containing fibroblasts, immune cells, adipocytes, muscle fiber. Four panels summarize drivers of SC dysfunction: vascular dysfunction—reduced VEGF/Ang1 and HGF/IGF signaling plus endothelial senescence ( ↑ IL‑6, MCP‑1, ROS) creating an inhibitory niche; SASP—senescent cells secrete MCP‑1, TGF‑β and IL‑6, recruit inflammatory cells and activate Smad/JAK‑STAT3 to drive fibrosis, limit proliferation and trigger aberrant differentiation; epigenetic alterations—MyoD/myogenin hypermethylation, p16INK4a demethylation and miRNA shifts ( ↑ miR‑34/29, ↓miR‑489/1) reduce myogenesis and enforce arrest/quiescence; immune dysregulation—↑M1/ ↓ M2 macrophages sustain TNF‑α/IL‑1β inflammation, inhibit Notch and promote SC apoptosis and failed repair

The senescence-associated secretory phenotype (SASP) is a key characteristic of senescent cells, involving increased secretion of inflammatory factors, chemokines, proteases, and growth factors (Fig. 4). In aging skeletal muscle, SCs and other senescent cells (e.g., fibroblasts, immune cells) express SASP, collectively creating an inhibitory inflammatory microenvironment.^116^ During aging, the regulatory balance of various secreted factors is completely disrupted; persistent activation of inflammatory cytokines damages satellite cells by activating downstream signaling pathways. Overactivated JAK/STAT3 signaling induces premature differentiation of satellite cells, leading to the continuous depletion of the quiescent stem cell pool; long-term excessive secretion of IL-6 also induces muscle fibrosis, hindering the muscle repair process. MCP-1 continuously recruits inflammatory cells to infiltrate muscle tissue, amplifying local inflammatory damage and further deteriorating the microenvironment.^54^ Furthermore, aging leads to functional dysregulation and imbalance of tissue regulatory factors: TGF-β is persistently overexpressed and activates the Smad signaling pathway, exacerbating tissue fibrosis and inhibiting satellite cell proliferation and activation; dysregulation and imbalance in VEGF expression prevent orderly regulation of angiogenesis, making it difficult to establish a vascular support system suitable for muscle regeneration.^98^ At the same time, an imbalance in the secretion ratio of MMPs and TIMPs leads to continuous disruption of the extracellular matrix structure, further limiting the survival space for satellite cells. SASP can form a self-amplifying pathological positive feedback loop by activating core transcription factors such as NF-κB and C/EBPβ, continuously reinforcing the inflammatory aging state and ultimately leading to progressive functional decline of satellite cells and an irreversible decrease in skeletal muscle regenerative capacity.^117^ Targeting the SASP and intervening in its vicious cycle using JAK inhibitors and senolytics can mitigate the adverse effects of chronic inflammation, restore satellite cell activity in aged individuals, and reshape the muscle microenvironment to support regeneration.^118^

### Epigenetic landscape of the aged niche

In addition to being driven by intrinsic cellular factors, the epigenetic remodeling of satellite cells is even more strongly regulated by the external microenvironment of aging skeletal muscle. Continuous stimulation from external signals—such as chronic inflammation, SASP secretion, and extracellular matrix fibrosis—induces satellite cells to develop a stable epigenetic profile characteristic of aging, which cannot be fully reversed by correcting the cells’ own mitochondrial metabolic defects alone.^119^ In the aging microenvironment, macrophages, fibro-adipogenic progenitor cells (FAPs), and aged myocytes continuously release SASP components such as CCL2, CCL7, IL-6, TGF-β, and other SASP components. These signals are amplified by the CD36 lipid transport receptor, leading to sustained activation of core transcriptional pathways such as NF-κB and SMAD3. This process rewrites the transcriptional landscape of satellite cells through multiple levels—including DNA methylation, histone modifications, and non-coding RNA—thereby shaping an epigenetic profile of aging that is distinct from endogenous cellular metabolic dysregulation.^120,121^ Reduced secretion of WISP1 by aged FAPs leads to impaired Akt signaling in MuSCs, affecting their ability to proliferate and undergo asymmetric division; Meanwhile, aged MuSCs themselves activate NF-κB through H3K27me3 erosion, thereby secreting IL-6 and Spp1, which promote the fibrotic differentiation of FAPs.^122^ When MuSCs detect these paracrine inflammatory signals, they are translated into intracellular epigenetic changes, creating a vicious cycle of inflammation, epigenetic erosion, and functional decline. As the central mediator of the aging microenvironment, the inflammatory microenvironment primarily reshapes the global distribution patterns of histone modifications in satellite cells through two mechanisms: disrupting the supply of substrates to modifying enzymes and causing direct oxidative damage. Under high concentrations of inflammatory factors characteristic of the aging niche, the expression of the methyltransferase Kmt5a is downregulated, leading to widespread erosion of the key inhibitory marker H4K20me1 in the quiescent state and increasing cellular sensitivity to ferroptosis.^119^ At the same time, the microenvironment continuously activates the NF-κB signaling pathway, leading to a massive loss of the H3K27me3 repressive mark in the Nfkb1 gene region of satellite cells. This results in a significant increase in chromatin accessibility, leading to the massive accumulation of NF-κB protein, which targets the IL6 and Spp1 gene enhancers and drives their high expression; The increased secretion of IL-6 and the bone bridge protein Spp1 further stimulates excessive proliferation of stromal FAPs and massive collagen synthesis, exacerbating the fibrotic microenvironment of the muscle and forming a positive feedback loop of histone disorganization and matrix stiffening.^122^ The aging microenvironment also depletes intracellular NAD⁺ reserves, inhibits the deacetylase activity of sirtuins, disrupts the distribution of activator marks such as H3K4ac and H3K9ac, and leads to sustained abnormal transcription of non-specific inflammatory and pro-fibrotic genes, thereby completely dismantling the chromatin landscape specific to the satellite cell lineage. ATAC-seq (chromatin accessibility sequencing) results confirm that, under the aging microenvironment, overall chromatin accessibility in satellite cells decreases, while the accessibility of promoters for inflammation- and fibrosis-related genes specifically increases; this chromatin remodeling pattern is entirely driven by the NF-κB and STAT pathways within the SASP.^120^

In addition, changes in histone modifications are closely associated with metabolic remodeling. Alterations in nutritional signaling and oxygen tension within the aging microenvironment can affect the availability of metabolites, thereby regulating the activity of epigenetic enzymes. Metabolites produced by fatty acid oxidation can influence the supply of acetyl-CoA, thereby regulating histone acetylation levels; meanwhile, α-ketoglutarate, an intermediate in the tricarboxylic acid cycle, serves as a key cofactor for histone demethylases, and changes in its levels can affect the methylation status of DNA and histones. This metabolic-epigenetic cross-regulation allows changes in the microenvironment to profoundly influence the epigenome of MuSCs through metabolic pathways.^83^ NF-κB and TGF-β in the aging microenvironment are the core upstream exogenous signals that regulate the reprogramming of the satellite non-coding RNA network. Unlike the unidirectional regulatory model of mitochondrial ROS, the synergistic action of multiple inflammatory pathways can stably modulate the overall abundance of microRNAs, long non-coding RNAs, and circular RNAs, thereby establishing a post-transcriptional bridge that translates microenvironmental signals into cellular fate decisions. Under continuous stimulation from the aging inflammatory niche, pro-aging microRNAs such as miR-29 and miR-34 are significantly upregulated. These two microRNAs specifically inhibit the epigenetic modification genes DNMT3A and SIRT1, simultaneously disrupting the homeostasis of methylation and the balance of histone acetylation, thereby doubly inhibiting the proliferative capacity of satellite cells; whereas miR-1, which maintains quiescence and orderly differentiation, and miR-489—which maintain quiescence and orderly differentiation—decreased significantly, directly leading to premature and abnormal activation of satellite cells and disruption of the differentiation program.^119^ TGF-β signaling in the microenvironment induces an imbalance in the expression of lncRNAs such as H19 and MEG3; abnormally enriched transcripts recruit various epigenetic modification complexes to stem cell-associated gene regions, promoting H3K27me3 deposition and stabilizing the silencing of genes related to self-renewal; Disrupted circRNA expression, in turn, disrupts the endogenous ceRNA competitive regulatory network, amplifying the effects of various pro-aging molecules, and multiple non-coding RNAs act synergistically to consolidate the senescence phenotype in satellite cells.^122^ TGF-β signaling in the microenvironment induces an imbalance in the expression of lncRNAs such as H19 and MEG3; abnormally enriched transcripts recruit various epigenetic modification complexes to stem cell-associated gene regions, promoting H3K27me3 deposition and stabilizing the silencing of genes related to self-renewal; Disrupted circRNA expression, in turn, disrupts the endogenous ceRNA competitive regulatory network, amplifying the effects of various pro-aging molecules, and multiple non-coding RNAs act synergistically to consolidate the senescence phenotype in satellite cells.^120^ The asynchronous transplantation experiment directly demonstrates the dominant role of the microenvironment in shaping the epigenetic profile of satellite cells; approximately 46.6%–53.6% of aging-associated differentially expressed genes were restored to levels close to those of young cells, indicating that aging-related transcriptomic changes are largely driven and maintained by signals from the external microenvironment. The remaining “irreversible” genes that did not revert showed a stronger correlation with changes in chromatin accessibility and DNA methylation, suggesting that stable epigenetic modifications may be key factors limiting transcriptomic plasticity.^123^

### Immune cell dysfunction and altered macrophage polarization

In young, healthy muscle tissue, macrophages are highly plastic and can dynamically switch between the pro-inflammatory M1 phenotype and the anti-inflammatory, reparative M2 phenotype in response to signals from the microenvironment, thereby orderly mediating the clearance and repair processes following injury.^124^ However, aging completely disrupts this orderly immune regulatory process. Due to persistent, low-grade chronic inflammation in aging muscle tissue, levels of key pro-inflammatory factors such as TNF-α, IL-1β, and IL-6 remain elevated, causing macrophages to become excessively biased toward the pro-inflammatory M1 phenotype and preventing them from undergoing the normal transition to the reparative M2 phenotype.^125^ Persistently activated M1 macrophages secrete large amounts of pro-inflammatory cytokines, which can both directly induce apoptosis in satellite cells and, via the NF-κB pathway, cause premature differentiation or senescence, gradually depleting the body’s limited stem cell reserves.^126^ In addition, the aging inflammatory microenvironment disrupts intercellular communication between macrophages and satellite cells, leading to impaired Notch signaling and blocked receptor-ligand binding. As a result, the macrophages’ supportive role in satellite cell self-renewal is significantly weakened, further exacerbating muscle regeneration defects.^127^

### Impaired angiogenesis and vascular dysfunction

With aging, skeletal muscle vascular network undergoes significant degeneration: reduced capillary density and endothelial cell dysfunction directly cause insufficient oxygen/nutrient supply for SCs and decreased supportive paracrine factor production. Studies indicate that in older people, skeletal muscle expression of pro-angiogenic factors like VEGF and angiopoietin-1 decreases, while anti-angiogenic factors like thrombospondin-1 increase, exacerbating vascular network degeneration.^128^ Dysfunctional senescent endothelial cells exhibit pro-inflammatory properties, secreting inflammatory mediators like IL-6 and MCP-1, contributing to inhibitory inflammatory microenvironment. Elevated oxidative stress levels from increased ROS production in these cells further impair SC function.^129^ Notably, bidirectional communication exists between SCs and endothelial cells: SCs themselves secrete multiple angiogenic factors regulating vascular formation. Age-related SC function decline weakens this positive feedback loop, creating a vicious cycle where diminished vascular support reduces SC function, which further lowers angiogenic capacity.^50^

## Intervention strategies and research progress

### Nutritional intervention: protein supplementation and calorie restriction

**Fig. 5:**
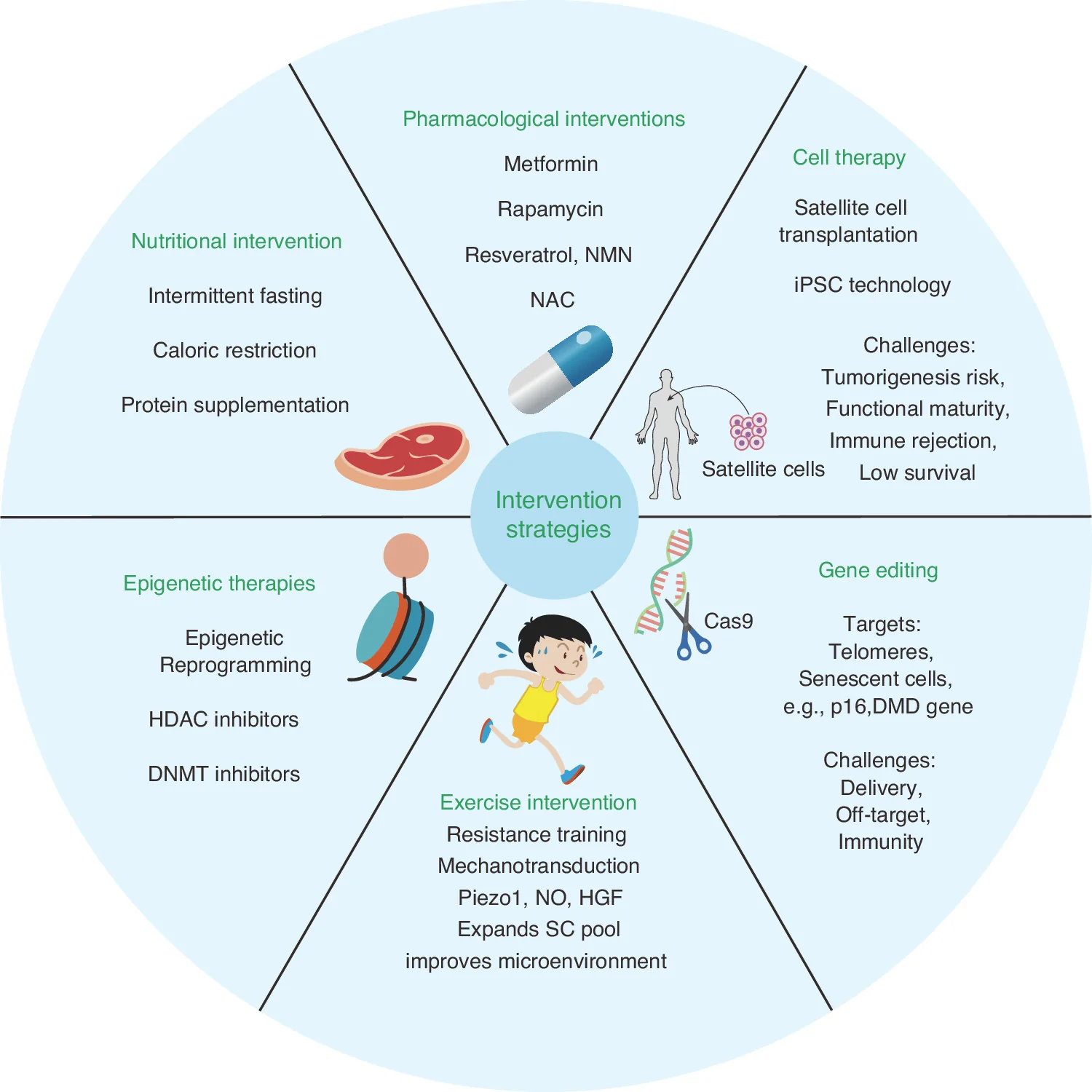
Intervention strategies for promoting muscle regeneration and delaying muscle decline. Nutritional interventions (intermittent fasting, caloric restriction, protein supplementation); Pharmacological interventions (metformin, rapamycin, NMN, resveratrol, NAC); Cell therapies (satellite cell transplantation, iPSC technologies)—challenges include tumorigenic risk, incomplete functional maturation, immune rejection, and low survival; Gene editing (Cas9-based approaches targeting telomeres or clearing senescent cells); Epigenetic therapies (reprogramming, HDAC and DNMT inhibitors); Exercise interventions (resistance training and mechanotransduction via Piezo1, NO, HGF) which expand the satellite cell pool and improve the muscle microenvironment

Nutritional interventions are crucial for maintaining SC function and counteracting muscle aging.^130^ Protein supplementation and caloric restriction have distinct yet significant effects. Protein supplementation supports SC function by providing anabolic substrates and activating key signaling pathways: moderately increasing protein intake, particularly high-quality protein, provides necessary amino acids for muscle protein synthesis (Fig. 5). Branched-chain amino acids like leucine effectively activate mTOR signaling, promoting SC proliferation and differentiation.^131^ This is particularly important in aging muscle due to “anabolic resistance”—reduced responsiveness to amino acid stimulation in older individuals, requiring higher protein doses to achieve comparable anabolic effects to younger adults.^132^ Protein intake exhibits a dose-response relationship with SC function: consuming 20–30 grams of high-quality protein (containing ~2–3 grams leucine) per meal maximally stimulates muscle protein synthesis rates, providing a scientific basis for elderly protein supplementation protocols.^133^ Whey protein, with rapid digestion and high leucine content, demonstrates unique advantages in promoting SC activation and muscle regeneration.^134^ Unlike protein supplementation’s anabolic effects, caloric restriction (CR) positively influences SC function by activating cellular stress resistance and autophagy mechanisms.^135^ Long-term moderate CR (typically reducing caloric intake by 25%–30%) improves multiple age-related biomarkers and delays sarcopenia onset. Molecularly, CR protects SCs from age-related functional decline by reducing insulin/IGF-1 signaling activity and activating longevity-associated pathways like AMPK and sirtuins (particularly SIRT1), also enhancing autophagy capacity and antioxidant defense systems.^136^ Though seemingly contradictory, protein supplementation and CR may synergistically promote SC health through distinct mechanisms. New dietary patterns like intermittent fasting or time-restricted eating aim to combine benefits of both by limiting food intake to specific periods while ensuring adequate protein consumption; preliminary studies suggest these interventions can enhance muscle mass and function in older adults.^137^

In terms of efficacy comparisons and clinical limitations, nutritional intervention is the safest, most universally applicable, and non-invasive foundational intervention among all intervention strategies. It is suitable for long-term use by healthy individuals of all ages and patients with mild sarcopenia; however, when used as a standalone intervention, its efficacy is limited, and it cannot reverse severe muscle aging or muscle satellite cell depletion.^138,139^ At the clinical translation level, this strategy faces significant challenges regarding individual customization and dose control. Specifically, digestive and absorptive functions vary greatly among older adults, and uniform standards for protein and calorie intake cannot accommodate individuals with different constitutions. In the elderly population, the secretion of gastric protease and pancreatic enzymes is 45%–55% lower than in young and middle-aged adults, and the digestion and absorption rate of conventional whey protein ranges from only 43.5%–52.8%, with individual absorption differences reaching up to a twofold variation; thus, a fixed intake dose struggles to meet the diverse needs of different older adults.^140^ In addition, when caloric intake is significantly reduced and protein intake is insufficient, elderly participants experience skeletal muscle loss in the short term; intermittent fasting may deplete long-lived plasma cells in the bone marrow, leading to a decrease in the secretion of long-acting antibodies, which in turn may increase the risk of respiratory infections.^141^ This suggests that excessive protein intake increases the metabolic burden on the kidneys in older adults, while excessive calorie restriction can lead to side effects such as malnutrition, weakened immunity, and temporary loss of muscle mass. At the same time, most existing studies are short-term observational trials; clinical trials on nutritional interventions for sarcopenia have an average duration of only 8–24 weeks, and less than 18% of studies include follow-up periods exceeding 1 year, making it difficult to ensure adherence to long-term dietary interventions.^142^ Furthermore, the positive effects of dietary interventions can only maintain homeostasis; they cannot reverse the aging and damage of muscle satellite cells at the genetic or cellular level, and therefore require synergistic effects from other approaches, such as exercise and medication.

### Exercise intervention: resistance training and mechanical stimulation

Resistance training is currently recognized as the most effective physiological intervention for activating SCs and promoting muscle regeneration. Mechanical loading deforms myofibrillar membrane and ECM, activating SCs via integrin-mediated mechanotransduction involving coordinated action of mechanosensitive ion channels (e.g., Piezo1), local growth factor release (e.g., HGF, FGF2), and nitric oxide (NO) signaling, collectively triggering SC transition from quiescence to activation.^143^ SC activation by resistance exercise is intensity-dependent: high-intensity exercise causes more significant microdamage, activating more SCs via inflammatory pathways.^144^ Damaged fibers release cytokines and chemokines recruiting immune cells like macrophages to injury sites; these cells promote SC proliferation and differentiation via factors like IL-6.^27^ Moderate inflammation is essential for regeneration, whereas chronic low-grade inflammation in aged muscle inhibits SC function; resistance exercise may benefit by shifting inflammatory state from chronic to acute.^125^ Long-term resistance training adaptively expands the muscle satellite cell pool: After several months of regular training, the number of muscle satellite cells surrounding muscle fibers increased significantly in both young and older subjects, and this increase was positively correlated with muscle hypertrophy. A standardized resistance training program lasting 12–16 weeks induced substantial proliferation of satellite cells specific to muscle fiber subtypes; the total number of Pax7-positive satellite cells increased by 22%–34% in young adults (ages 18–35), while the increase in older adults (ages 65–80) reached 28%–48%, with the expansion of Type II fast-twitch muscle fiber satellite cells far exceeding that of Type I slow-twitch muscle fibers. This long-term accumulation of satellite cells is strongly positively correlated with increases in muscle fiber cross-sectional area and overall muscle hypertrophy.^145^ Expansion may occur through multiple mechanisms: suppressed apoptosis, enhanced self-renewal capacity, and promoted SC-basement membrane adhesion.^146^ Resistance exercise also positively influences SC microenvironment: promoting angiogenesis improves blood supply and nutrient delivery; modulating ECM composition provides more suitable anchoring substrates; reducing fat infiltration and fibrosis creates a more regenerative and supportive microenvironment,^147^ collectively favoring SC function.

Among all intervention strategies, resistance training offers the best overall cost-effectiveness and clinical efficacy. It is currently the only first-line intervention for muscle aging recommended by clinical guidelines, with significantly better outcomes than nutritional intervention alone, and without the safety risks associated with drug or cell therapies.^148^ Its core advantage lies in its ability to bidirectionally regulate the function of muscle satellite cells across multiple levels—including physiological, microenvironmental, and cellular—in a bidirectional manner. Regular resistance training can steadily expand the body’s pool of muscle satellite cells and promote the continuous regeneration of damaged skeletal muscle. The mechanical stimulation resulting from training can also gradually reduce the accumulation of chronic inflammation and tissue fibrosis within aging muscles, ultimately leading to a long-term, sustained improvement in muscle function.^149^ However, this strategy has inherent limitations in its applicability and upper limits on efficacy that are difficult to avoid. Elderly individuals with disabilities, those with osteoarthritis, and those with severe systemic frailty lack the physical capacity to independently perform regular resistance training; mechanical stimulation cannot effectively act on skeletal muscle tissue, making it difficult for such interventions to yield practical benefits. Epidemiological survey data show that among severely frail older adults aged 75 and older, less than 20% are able to independently perform standardized strength training.^150^ For patients with hereditary muscular dystrophy and individuals with severe sarcopenia caused by extensive depletion of myosatellite cells, exercise alone can only slow the overall progression of muscle decline; it cannot reverse the pathological skeletal muscle damage and stem cell aging that have already occurred.^151^ The implementation of exercise interventions requires strict control of training load and safety parameters. High-intensity training that exceeds the body’s tolerance limits can induce a persistent, low-grade inflammatory response in skeletal muscle; excessive mechanical damage to muscle fibers can downregulate the proliferation and differentiation of muscle satellite cells, thereby impairing muscle regeneration capacity. At present, there are no universally accepted or widely applicable standards for determining individualized training intensities for older adults worldwide. Significant differences exist among participants in terms of baseline muscle condition and comorbidities, resulting in a wide range of individual variation in the final outcomes of interventions. Results from existing meta-analyses suggest that, under training of equal intensity, the individual variation in muscle strength improvement among older adults can reach up to 1.8-fold.^152^

### Pharmacological interventions: antioxidants and signaling pathway modulators

Chronic inflammation and oxidative stress are key factors in SC functional decline and muscle aging; intervention strategies targeting these processes, such as anti-inflammatory and antioxidant approaches, are important research avenues for alleviating sarcopenia and improving muscle regenerative capacity. N-acetylcysteine (NAC) is extensively studied as an antioxidant prodrug enhancing intracellular glutathione (GSH) synthesis.^153^ GSH is the most important endogenous antioxidant, participating in ROS scavenging and redox homeostasis maintenance. In aged muscles, elevated oxidative stress and decreased GSH/GSSG ratio impair SC function; studies indicate NAC supplementation (typically 600–1200 mg daily) significantly reduces oxidative stress levels in elderly muscles and enhances SC proliferation and differentiation capacity.^154^ In animal models, NAC treatment mitigates age-related muscle atrophy and enhances contractile function, likely through multiple pathways: direct ROS scavenging, reduced mitochondrial oxidative damage, inhibited NF-κB inflammatory pathway activation, and regulated apoptosis signaling.^155^ Metformin, a classic hypoglycemic agent, has recently been found to have multifaceted effects beyond glucose control, including anti-inflammatory, antioxidant, and anti-aging properties.^156^ In muscle aging and SC function maintenance, metformin exerts protective effects through multiple mechanisms: core mechanism involves activating AMPK by inhibiting mitochondrial complex I, creating mild ATP deficiency.^157^ AMPK activation regulates energy metabolism and suppresses mTORC1 signaling, mitigating age-related mTORC1 overactivation, contributing to enhanced metabolic flexibility and stress resistance. Furthermore, metformin suppresses NF-κB signaling, reducing pro-inflammatory factors like TNF-α and IL-6, mitigating chronic inflammation inhibition on SCs.^158^ Metformin also enhances cellular antioxidant defenses by regulating Nrf2 pathway: it promotes Nrf2 nuclear translocation, increasing expression of antioxidant enzymes like heme oxygenase-1 (HO-1) and quinone oxidoreductase 1 (NQO1), mitigating oxidative stress damage to SCs.^159^

Beyond traditional anti-inflammatory/antioxidant agents, small-molecule drugs targeting specific signaling pathways offer more precise intervention strategies for SC aging, directly targeting key nodes regulating cellular metabolism, stress resistance, and lifespan, making them promising candidates for delaying or reversing age-related SC functional decline. mTOR inhibitors like rapamycin and its analogs (rapalogs) are among the most extensively studied anti-aging drugs.^160^ Excessive mTORC1 activation is associated with multiple age-related diseases including sarcopenia; studies show intermittent low-dose rapamycin administration suppresses mTORC1 signaling, mitigates age-related SC dysfunction, and enhances muscle regenerative capacity by inducing autophagy, improving protein homeostasis, reducing cellular senescence, and suppressing inflammatory responses.^161^ However, complete mTOR inhibition can negatively affect muscle mass, so optimizing dosage and regimen is crucial. AMPK activators like AICAR and metformin exert protective effects by mimicking energy-restricted states.^162^ AMPK activation promotes mitochondrial biogenesis, enhances autophagy, improves glucose metabolism, and reduces oxidative stress. Studies reveal AMPK activator treatment improves SC function in aged animals, enhancing proliferation and differentiation capacity, likely related to AMPK-regulated metabolic reprogramming enabling SCs to better adapt to age-related energy metabolism changes.^163^ Sirtuin activators like resveratrol and SRT1720 have anti-aging properties by mimicking calorie restriction benefits:^164^ sirtuins are NAD^+^-dependent deacetylases regulating metabolism, stress responses, genomic stability, and aging. SIRT1 activation promotes mitochondrial function, enhances antioxidant defenses, improves DNA repair capacity, and suppresses inflammatory responses. Studies show NAD^+^ precursor supplementation (e.g., nicotinamide mononucleotide, NMN) or SIRT1 activators can improve SC function in aged animals and delay muscle aging.^165^ These pathways interact complexly: e.g., AMPK activation increases NAD^+^ levels, activating sirtuins; SIRT1 deacetylates and activates metabolic transcription factors, creating positive feedback. Therefore, targeting multiple pathways simultaneously may produce synergistic effects and offer more effective strategies for intervening SC senescence.^166^

Pharmacological interventions can precisely target signaling pathways associated with age-related disorders, offering greater overall precision than nutritional or exercise interventions. These interventions can improve abnormally elevated levels of oxidative stress and chronic inflammation in individuals with moderate sarcopenia, resulting in a clear improvement in muscle function among this elderly population. However, pharmacological regulation can only correct imbalances at the cellular metabolic level; its overall efficacy falls short of that of cell transplantation and gene editing techniques, and it cannot achieve the fundamental repair and regenerative remodeling of muscle satellite cells. The clinical translation process for various anti-aging small-molecule drugs has always been fraught with controversy, and the overall barriers to implementation remain significant. The core factors limiting their clinical application center on the safe dosage range, differences in efficacy across species, and the side effects caused by long-term administration. The vast majority of drugs with anti-aging potential exhibit diametrically opposed physiological effects depending on the dosage. For example, low-dose rapamycin can moderately induce cellular autophagy to slow the aging process in muscles, but when the dosage exceeds the safe range, it continuously inhibits skeletal muscle protein synthesis, ultimately leading to muscle atrophy.^167^ Excessive supplementation with N-acetylcysteine can disrupt the body’s natural redox balance, and the excretion of excess drug components through the liver and kidneys can also increase the burden on these organs. At present, there is no globally standardized dosage reference system specifically applicable to the elderly. The skeletal muscle-protective effects demonstrated by most small-molecule drugs can only be consistently replicated in aging rodent animal models; when the same dosing regimens are applied in human clinical trials, the drugs often exhibit a significant decline in efficacy or even become completely ineffective. A meta-analysis of 167 basic anti-aging studies revealed that more than 70% of drug candidates targeting muscle aging failed to replicate the positive effects observed in animal experiments.^168^ While the conditions for establishing animal models of aging are relatively simple, the aging process of human muscle is regulated by a combination of multiple external factors, including age, underlying chronic diseases, and systemic metabolic status; this complex internal environment significantly reduces the efficacy of interventions targeting a single molecular target. Furthermore, long-term, continuous use of widely used anti-aging drugs such as metformin and rapamycin can cause a variety of adverse reactions in the body. Long-term intake of metformin can reduce the gut’s ability to absorb vitamin B12 by about 30%, and some users may experience recurrent gastrointestinal discomfort, such as bloating, pain, and diarrhea.^169^ Long-term use of rapamycin can easily lead to problems such as oral mucosal ulcers, dyslipidemia, and a downregulation of the body’s immune response.^170^ Healthy older adults do not need to rely on medications for preventive anti-aging interventions; various targeted drugs are only suitable as adjunctive treatments for individuals with sarcopenia accompanied by pathological damage.

### Cell therapy: satellite cell transplantation and iPSCs technology

Satellite cell transplantation is a promising treatment for severe muscle injuries and degenerative diseases like Duchenne muscular dystrophy.^171^ Studies indicate transplanting SCs from young donors into aged recipient muscles significantly enhances muscle regenerative capacity, partially restoring youthful phenotype.^172^ This approach enables transplanted cells to directly participate in regeneration and may improve native SC function via paracrine mechanisms.^173^ However, exogenous SC transplantation faces challenges: primary concern is immune rejection—allogeneic transplantation triggers host immune system recognition and attack, clearing transplanted cells.^174^ Immunosuppressive drugs can mitigate rejection but have severe side effects long-term; researchers explore strategies like MHC-matched donor cells, immune-privileged microenvironment construction, and reducing cell immunogenicity via gene editing.^19^ Secondly, cell survival and integration efficiency remains low: over 90% transplanted SCs die within 1 week, only a small proportion colonizing and functioning.^175^ Extensive cell death associates with mechanical injury during transplantation, hypoxic-ischemic environments, and host inflammatory responses; improved transplantation strategies (e.g., biomaterial scaffolds for 3D support, growth factor delivery systems, optimized cell preparation methods) significantly enhance survival rates.^176^ Additionally, age-related microenvironmental barriers exist: aging muscle microenvironment may suppress transplanted cell function through chronic inflammation, abnormal ECM deposition, and vascular network degeneration.^100^ Thus, successful cell transplantation may require microenvironment remodeling—e.g., reducing inflammation and improving vascularization via pharmacological interventions—creating a more favorable environment for transplanted cell survival.^177^

Induced pluripotent stem cell (iPSC) discovery provides a novel cellular source for muscle tissue regenerative medicine. iPSC technology enables somatic cells (e.g., skin fibroblasts, blood cells) reprogramming into pluripotency, then differentiation into various cell types including skeletal muscle lineage,^178^ avoiding embryonic stem cell ethical concerns and enabling personalized therapy using patient’s own cells. Significant progress has been made in differentiating iPSCs into skeletal muscle cells: mimicking embryonic skeletal muscle development, researchers developed efficient differentiation protocols converting iPSCs into Pax7^+^ skeletal muscle progenitor cells with myogenic potential in vitro and in vivo.^179^ Modulating specific signaling pathways (e.g., Wnt, BMP, Notch) can obtain myogenic cells at different developmental stages meeting diverse therapeutic needs.^180^ In disease modeling and drug screening, iPSC technology offers distinct advantages: iPSCs derived from patients with muscular diseases can differentiate into disease-specific muscle cells, providing a vital platform for investigating disease mechanisms and testing potential treatments.^181^ For example, iPSC models from Duchenne muscular dystrophy patients enabled identification of multiple therapeutic compounds.^182^ However, iPSC clinical application still faces safety challenges like tumor formation risk and insufficient functional maturity: undifferentiated or partially reprogrammed cells may form teratomas;^183^ mitigating this risk requires enhancing differentiation purity, developing safer reprogramming methods (e.g., using small molecules instead of genome-integration factors), and establishing effective cell purification strategies.^184^ Despite challenges, iPSC technology holds great promise in muscle regenerative medicine; as differentiation techniques refine and safety improves, iPSC-derived myogenic cells are expected to provide novel solutions for cell therapy in muscle degenerative diseases.^185^

Among the six major interventions for muscle aging, cell therapy offers the greatest potential for tissue regeneration and the most effective reversal of pathological damage. In terms of improving physical function in cases of severe skeletal muscle damage caused by hereditary myopathies and end-stage sarcopenia, its efficacy comprehensively surpasses that of nutritional interventions, exercise interventions, and conventional pharmacological treatment regimens. This intervention strategy replenishes the body’s depleted pool of muscle satellite cells at the cellular level and comprehensively repairs the structure of damaged skeletal muscle tissue, making it the most effective high-end treatment currently available. Conversely, this technology presents the highest overall R&D barriers and clinical operational complexity among all intervention methods. Its high overall application costs and highly limited applicability preclude its use for the routine prevention and control of physiological muscle aging in the general population; it is suitable only for targeted treatment of severe degenerative muscle disorders. Throughout the entire process of allogeneic myosatellite cell transplantation, the transplanted cells are recognized and eliminated by the host’s immune system; immune rejection significantly reduces the survival rate and engraftment efficiency of the transplanted cells. Statistical data from multiple preclinical trials on skeletal muscle transplantation show that the overall survival rate of myosatellite cells implanted into recipient muscle tissue without the aid of a biomaterial carrier is less than 10% within 1 week; the vast majority of transplanted cells undergo apoptosis due to the combined effects of ischemia, hypoxia, and local inflammation.^186^ Aging skeletal muscle exhibits pathological changes such as chronic inflammatory infiltration, abnormal deposition in the extracellular matrix, and degeneration of the vascular network. This adverse microenvironment continuously inhibits the proliferation and differentiation of transplanted stem cells. Relying solely on cell infusion is insufficient to restore a local tissue microenvironment conducive to stem cell survival, and long-term muscle regeneration outcomes will show a gradual decline over time.^187^ Furthermore, the process of large-scale in vitro expansion and preparation of myosatellite cells is plagued by an unavoidable loss of stem cell properties; specifically, during continuous passaging, the cells spontaneously differentiate into myoblasts, gradually losing their core stem cell attributes of resting reserve and self-renewal. After four to five consecutive passages of human myosatellite cells, the proportion of cells exhibiting stem cell characteristics drops precipitously; cells that have undergone ten passages are virtually incapable of achieving long-term skeletal muscle remodeling when transplanted into the body.^188^ Prolonged in vitro culture also increases the proportion of senescent cells, and the accumulation of senescent cells further impairs the overall efficacy of transplantation. Myogenic precursor cells derived from induced pluripotent stem cells via directed differentiation also suffer from a lack of maturity; these cells generally retain the cellular phenotype of the embryonic and neonatal stages, and their contractile protein subtypes, energy metabolism patterns, and tissue regeneration rhythms all differ significantly from those of endogenous muscle satellite cells in adult organisms. Once transplanted into adult muscle tissue, these cells exhibit low functional integration efficiency and struggle to meet the physiological demands of maintaining homeostasis and repairing damage in adult skeletal muscle.^189^ Even if the recipient’s microenvironment can slowly promote cell maturation, the overall transformation process is protracted, and the cells’ ultimate function still cannot match that of natural adult muscle satellite cells.

### Gene editing: CRISPR-Cas9 applications for aging intervention

CRISPR/Cas9 gene editing technology provides a powerful tool for precisely targeting aging-related genes, offering new hope for treating age-related diseases. For intervening SC senescence, CRISPR primarily targets several areas: repairing telomere maintenance-related genes—editing telomerase reverse transcriptase (TERT) gene enhances telomerase activity, delaying telomere attrition;^190^ preclinical studies suggest this intervention could extend cellular replicative lifespan and enhance stem cell function.^191^ Clearing senescent cells—targeting key senescence marker p16INK4a, strategies like promoter-driven prodrug convertase expression can selectively activate apoptosis signaling in senescent cells,^192^ eliminating harmful cells and improving tissue microenvironment. In muscle-specific applications, CRISPR successfully treats genetic disorders like Duchenne muscular dystrophy (DMD):^193^ editing patient-derived iPSCs in vitro or muscle tissue in vivo restores dystrophin expression and improves muscle function, providing insights for gene editing in age-related muscle decline treatment. However, CRISPR clinical application still faces challenges: delivery efficiency, off-target effects, and immunogenicity.^194^ Efficient and specific delivery of editing components to SCs remains critical; novel delivery systems like optimized adeno-associated virus (AAV) vectors and lipid nanoparticles are actively investigated.^195^

However, the clinical application of CRISPR still faces numerous challenges. Existing research has clearly identified that delivery efficiency, off-target effects, and immunogenicity are the core bottlenecks limiting the clinical translation of CRISPR-Cas9; these issues are particularly pronounced in targeted interventions for muscle aging.^196^ Although mainstream AAV vectors are widely used for in vivo gene delivery, they have a fixed packaging capacity limit, with the length of exogenous gene fragments they can accommodate not exceeding 4700 base pairs.^196^ The complete Cas9 coding sequence, combined with the necessary promoters and regulatory elements, often exceeds this capacity limit; researchers typically employ a dual-vector split delivery strategy. This approach relies on a single muscle cell simultaneously taking up both vectors, but the co-transfection efficiency remains at a low level of only 10%–30%, which directly results in low overall gene editing efficiency in aged skeletal muscle.^197^ Most adults possess natural neutralizing antibodies against adeno-associated viruses, with an overall population seroprevalence rate exceeding 70%. This pre-existing immune response directly blocks vector delivery and hinders repeat dosing in patients, making it impossible to achieve long-term, stable genetic modification of myosatellite cells.^198^ Although lipid nanoparticles circumvent the problem of immune priming associated with viral vectors, they lack the ability to specifically target and enrich quiescent muscle satellite cells, and their cell-specific delivery efficiency falls short of the requirements for precise anti-aging interventions. Furthermore, the exogenous Cas9 protein is inherently immunogenic and can activate both innate and adaptive immune responses in humans; 58% of healthy individuals have anti-SpCas9 antibodies, and 67% harbor SpCas9-specific T cells; 78% of the population tested positive for SaCas9 antibodies, and 78% harbored SaCas9-reactive T cells.^199^ Edited myosatellite cells are easily recognized and eliminated by the body’s immune system, making it difficult to maintain stable, long-term gene modification effects. Even when using engineered high-fidelity Cas9 variants, low-frequency nonspecific genome editing events cannot be completely eliminated.^200^ Myosatellite cells are long-lived adult stem cells; their characteristic of continuous renewal and division throughout life leads to the gradual accumulation of a small number of off-target mutations, which continuously increase genomic instability and the potential risk of tumorigenesis. For anti-aging interventions aimed at improving health in the elderly, such long-term safety risks are difficult to assess through clinical benefit evaluations. As the most precise technological approach among all current interventions for muscle aging, gene editing can directly repair age-related genetic defects or mutations in disease-causing genes, thereby reversing pathological damage to skeletal muscle at the genetic level. Theoretically, its overall efficacy surpasses that of cell therapy and represents the core direction for the future of precision prevention and treatment of muscle aging. Leveraging the characteristics of site-specific genomic modification, gene editing has the potential to halt degenerative changes in muscle at their root cause and represents the intervention type that comes closest to a curative effect at the mechanistic level. However, the overall clinical translation of this technology requires overcoming complex and multifaceted practical obstacles, and various limiting factors collectively raise the barriers to its practical application. At this stage, the technology is not yet ready for routine intervention in sarcopenia among the general elderly population and can only be used for the targeted diagnosis and treatment of various rare hereditary myopathies.

### Epigenetic-targeted therapies: DNMT and HDAC inhibitors

Epigenetic modifications serve as the central mediators through which environmental signals regulate gene expression; interventions targeting DNA methylation and histone acetylation can reverse the abnormal epigenetic remodeling that occurs during the aging process.^201^ DNA methyltransferase inhibitors such as 5-azacytidine and decitabine can reduce genome-wide methylation levels. Multiple in vitro experiments confirm that low-dose treatment corrects aging-associated hypermethylation and restores youthful transcriptional profiles; in muscle satellite cell models, such agents boost proliferation and differentiation and delay cellular senescence.^73,202^ Histone deacetylase (HDAC) inhibitors likewise exhibit muscle aging regulatory potential. Compounds including valproic acid and curcumin suppress HDAC activity, open chromatin and upregulate myogenic genes. Data from aged muscle animal models demonstrate that HDAC inhibition elevates muscle differentiation-related transcripts and slows muscle mass loss.^41,203^ Distinct HDAC subtypes carry divergent regulatory roles in muscle regeneration: HDAC1/2 dominate cell cycle control, while HDAC4/5 mainly modulate myogenic differentiation, making subtype-selective inhibitors capable of more precise muscle-targeted epigenetic regulation.^204^ Various natural plant extracts also exert mild epigenetic regulatory effects. Resveratrol delays the muscle aging process by activating the SIRT1 protein; epigallocatechin gallate (EGCG) inhibits DNMT enzyme activity; and curcumin regulates histone modification levels. These natural substances offer a higher safety profile for long-term use and are suitable as long-term therapeutic approaches.^205^ In recent years, breakthroughs have been made in epigenetic reprogramming technology. Short-term induction of the four Yamanaka factors—OCT4, SOX2, KLF4, and c-MYC—can reverse age-related epigenetic changes while preserving the cells’ intrinsic identity. Animal studies have confirmed that reprogramming can restore the transcriptional characteristics of young cells and improve the physiological function of aged tissues, providing a new research direction for interventions targeting age-related muscle decline.^206^

The comprehensive therapeutic effects of epigenetic interventions fall between those of conventional anti-inflammatory and antioxidant small-molecule agents and gene-editing technologies. Relying on reversible genomic modification mechanisms, they do not cause permanent changes to DNA sequences and, compared to gene editing and stem cell transplantation protocols, present lower overall safety risks. and their regulatory precision also surpasses that of conventional anti-aging active substances. However, this class of interventions faces multiple insurmountable barriers to clinical translation and is the most difficult to predict in terms of implementation prospects among the six major muscle aging intervention strategies. Traditional broad-spectrum DNMT and HDAC inhibitors lack the ability to target modifications at a single site; upon administration, they simultaneously alter the methylation and acetylation levels of thousands of coding genes. This large-scale, genome-wide epigenetic remodeling can induce multi-level, unpredictable physiological disruptions in multiple organs and tissues of the elderly.^207,208^ As a low-intensity chemotherapy agent for hematologic malignancies, decitabine yields only modest regulatory benefits within a strictly defined range of tumor doses. Its use in elderly cancer patients is still associated with significant bone marrow toxicity. If its use were expanded to the general elderly population without malignant lesions for long-term anti-aging interventions, the risk of organ damage would significantly outweigh the minimal benefits in slowing aging.^209^

The comprehensive therapeutic effects of epigenetic interventions fall between those of conventional anti-inflammatory and antioxidant small-molecule agents and gene-editing technologies. Relying on reversible genomic modification mechanisms, they do not cause permanent changes to DNA sequences and, compared to gene editing and stem cell transplantation protocols, present lower overall safety risks. and their regulatory precision also surpasses that of conventional anti-aging active ingredients. However, this class of interventions faces multiple insurmountable barriers to clinical translation and is the technical approach with the most unpredictable implementation prospects among the six major muscle aging intervention strategies. Lack of safety and specificity are the core challenges limiting the widespread use of drugs targeting DNMTs and HDACs. First-generation broad-spectrum HDAC inhibitors (such as vorinostat) lack selectivity for various HDAC subtypes and have induced significant dose-limiting toxicities in clinical use, including hematologic adverse reactions, gastrointestinal symptoms, and cardiac events, which severely limit their long-term administration and the implementation of combination therapy regimens.^210^ Similarly, the mechanism of action of nucleoside analog DNMT inhibitors (such as decitabine) relies on incorporation during DNA replication. This not only leads to target-related toxicities such as bone marrow suppression but also, because it induces global rather than gene-specific DNA demethylation, carries the risk of inadvertently activating endogenous retroelements or oncogenes, which may trigger genomic instability.^211^ Limited efficacy and high interindividual heterogeneity constitute another major obstacle. In elderly patients with acute myeloid leukemia, even with monotherapy using demethylating agents, the median overall survival has not exceeded 1 year, and a significant proportion of patients exhibit primary resistance.^209^ In muscle aging models, the efficacy of HDAC inhibitors is highly dependent on cell type, differentiation stage, and the targeted HDAC subtype, which greatly limits their feasibility as a universal intervention.^212^ Shortcomings in drug delivery efficiency and pharmacokinetic performance are equally significant. Many epigenetic drugs have low oral bioavailability, short in vivo half-lives, and difficulty effectively crossing the blood-brain barrier; these characteristics severely limit their application in age-related diseases affecting the nervous system and solid organs.^207^ Furthermore, the widespread discrepancy between preclinical studies and clinical outcomes stems from the inability of in vitro models to accurately simulate the complex cellular heterogeneity, chronic inflammatory microenvironment, and dynamic plasticity of epigenetic regulatory networks found in aging tissues. Consequently, many intervention strategies that prove effective at the cellular or animal level fail to reproduce their expected therapeutic effects in human trials.^213^ Therefore, to advance epigenetically targeted therapies toward clinical application, there is an urgent need to develop highly selective HDAC subtype inhibitors, non-nucleoside DNMT-targeting molecules, or CRISPR-dCas9-based site-specific epigenetic editing tools,^214^ supplemented by precise patient stratification strategies and rational combination therapy regimens, with the aim of achieving safe, effective, and sustained anti-aging interventions.

We have outlined and listed classifications, key action mechanism, functions and their limitations of all above therapeutic strategies in Table 2.

**Table 2:** Intervention strategies for skeletal muscle aging: key mechanisms and clinical considerations

Strategy	Key mechanisms	Functions	Limitations
Nutritional intervention	Protein supplementation (leucine, whey); Caloric restriction (25%–30%); Intermittent fasting.	20–30 g protein/meal (2–3 g leucine) maximally stimulates MPS;^133^ CR (25%–30%) delays sarcopenia onset;^135^ Activates mTOR via leucine;^131^ Enhances autophagy via AMPK/SIRT1.^136^	Protein absorption only 43.5%–52.8% in elderly;^140^ Short-term muscle loss (0.5–1.0 kg) during CR;^141^ < 18% of studies have >1-year follow-up;^142^ Cannot reverse genetic-level SC damage.
Exercise intervention	Resistance training; Mechanotransduction (Piezo1, integrins); HGF/NO release.	12–16 weeks training: Pax7⁺ SCs ↑22%–34% (young) & ↑28%–48% (elderly);^145^ Muscle fiber CSA ↑ 15%–25%;^145^ Angiogenesis & ECM remodeling improved.^147^	<20% of >75-year-old frail elderly can perform standard training;^150^ Individual variation in strength gain up to 1.8-fold;^152^ High-intensity overload may induce chronic inflammation and impair SC function.
Pharmacological intervention	Metformin (AMPK activator); Rapamycin (mTOR inhibitor); NAC (antioxidant); NMN/Resveratrol (SIRT1 activators).	NAC (600–1200 mg/d) reduces oxidative stress and improves SC proliferation;^154^ Rapamycin induces autophagy and extends lifespan in animal models;^160^ NMN restores NAD⁺ levels and improves SC function.^165^	>70% preclinical benefits fail in human trials;^168^ Metformin reduces B12 absorption by ~30%;^169^ Rapamycin causes oral ulcers, dyslipidemia, immunosuppression;^170^ Narrow therapeutic window; dose-dependent muscle atrophy risk.^167^
Cell therapy	SC transplantation; iPSC-derived myogenic progenitors; Biomaterial scaffolds.	Directly replenishes SC pool; Paracrine rejuvenation of native SCs;^173^ Personalized therapy using patient-derived iPSCs;^178^ Young donor SCs enhance aged muscle regeneration.^172^	>90% transplanted SCs die within 1 week without biomaterial support;^175^ SCs lose stemness after 4–5 passages in vitro;^188^ Only <10% engraftment efficiency;^175^ Tumorigenic risk;^183^ Functional immaturity of iPSC-derived cells.^189^
Gene editing	CRISPR-Cas9; AAV/lipid nanoparticle delivery; Targeting TERT; p16INK4a; dystrophin.	Repairs senescence-related genes and corrects genetic defects;^190,193^ Selectively clears p16INK4a⁺ senescent cells;^192^ Restores dystrophin expression in DMD models.^193^	AAV dual-vector co-transfection efficiency only 10%–30%;^196^ ~ 70% population has pre-existing AAV neutralizing antibodies;^198^ > 58% have anti-SpCas9 antibodies; 67% harbor SpCas9-specific T cells;^199^ Off-target mutation rate 0.1%–1%;^200^ High cost and technical barriers.
Epigenetic therapy	DNMT inhibitors (5-azacytidine); HDAC inhibitors (valproic acid); Yamanaka factor reprogramming.	Low-dose 5-azacytidine restores proliferation in aged SC models;^202^ HDAC inhibitors upregulate myogenic genes and slow muscle loss in animal models;^203^ Partial reprogramming reverses age-related epigenetic changes.^206^	Broad-spectrum DNMT/HDAC inhibitors lack site specificity → global epigenetic remodeling and unpredictable systemic toxicity (e.g., bone marrow suppression);^209^ Low oral bioavailability and short half-life;^207^ High inter-individual heterogeneity; preclinical-to-clinical translation failure is common.^213^

## Conclusion and perspectives

The progressive functional decline of SCs lies at the core of skeletal muscle aging, driving the pathogenesis of sarcopenia. With advancing age, SCs exhibit intrinsic alterations—including telomere attrition, mitochondrial dysfunction, epigenetic drift, and dysregulation of key signaling pathways such as Notch/Wnt and TGF-β—that collectively impair their quiescence, activation, proliferation, and differentiation capacities. Externally, the aged niche exacerbates SC dysfunction through chronic inflammation, oxidative stress, aberrant extracellular matrix remodeling, immune cell dysregulation, and vascular decline. These multifactorial changes disrupt the finely tuned microenvironment necessary for muscle regeneration, leading to reduced myogenic potential, fibrous–adipose infiltration, and loss of muscle mass and strength. Importantly, interdisciplinary research integrating single-cell genomics, epigenetics, bioengineering, and immune profiling has substantially advanced our understanding of these mechanisms, highlighting the interplay between cell-autonomous deficits and non-cell-autonomous microenvironmental factors in muscle aging.

Future research should focus on translating these mechanistic insights into targeted interventions that restore SC function and rejuvenate the muscle niche. Promising strategies include combinatorial approaches: senolytics to eliminate aged SCs, epigenetic modulators (e.g., DNMT/HDAC inhibitors) to reverse dysregulated gene expression, and metabolic agents (e.g., AMPK/SIRT1 activators) to enhance bioenergetic efficiency. Cell-based therapies using edited iPSCs or rejuvenated SCs, coupled with biomaterial-supported delivery, may overcome limitations in cell survival and integration. Moreover, personalized interventions—incorporating nutritional optimization, structured exercise, and pharmacological treatments—should be tailored to individual epigenetic and metabolic profiles. Embracing multidisciplinary innovations, such as CRISPR-based gene editing, spatially resolved transcriptomics, and artificial intelligence-driven drug discovery, will be essential to develop effective therapies against sarcopenia and improve healthspan in the aging population.
